# Multi-Level Perception Systems in Fusion of Lifeforms: Classification, Challenges and Future Conceptions

**DOI:** 10.3390/s26020576

**Published:** 2026-01-15

**Authors:** Bingao Zhang, Xinyan You, Yiding Liu, Jingjing Xu, Shengyong Xu

**Affiliations:** 1 The Key Laboratory for the Physics and Chemistry of Nanodevices, Institute of Physical Electronics, Department of Electronics, Peking University, Beijing 100871, China; zhangbingao@pku.edu.cn (B.Z.); 2301213327@pku.edu.cn (Y.L.); 2 School of Electronic and Information Engineering, Beihang University, Beijing 100191, China; 22371065@buaa.edu.cn; 3 School of Integrated Circuits, Shandong University, Jinan 250010, China

**Keywords:** fusion of lifeforms, multi-level perception, neuroprosthetics, implantable sensors, brain–computer interface, sensory restoration, cognitive enhancement, human–machine integration

## Abstract

The emerging paradigm of “fusion of lifeforms” represents a transformative shift from conventional human–machine interfaces toward deeply integrated symbiotic systems, where biological and artificial components co-adapt structurally, energetically, informationally, and cognitively. This review systematically classifies multi-level perception systems within fusion of lifeforms into four functional categories: sensory and functional restoration, beyond-natural sensing, endogenous state sensing, and cognitive enhancement. We survey recent advances in neuroprosthetics, sensory augmentation, closed-loop physiological monitoring, and brain–computer interfaces, highlighting the transition from substitution to fusion. Despite significant progress, critical challenges remain, including multi-source heterogeneous integration, bandwidth and latency limitations, power and thermal constraints, biocompatibility, and system-level safety. We propose future directions such as layered in-body communication networks, sustainable energy strategies, advanced biointerfaces, and robust safety frameworks. Ethical considerations regarding self-identity, neural privacy, and legal responsibility are also discussed. This work aims to provide a comprehensive reference and roadmap for the development of next-generation fusion of lifeforms, ultimately steering human–machine integration from episodic functional repair toward sustained, multi-level symbiosis between biological and artificial systems.

## 1. Introduction

Human–machine integration has evolved from simple assistive tools to tightly coupled systems such as advanced prostheses [1], implantable sensors [2], and brain–computer interfaces (BCI) [3], achieving ever-higher spatial density, bandwidth, and long-term stability. Yet, many prevailing paradigms—often framed as human–machine interfaces (HMIs) or brain–machine interfaces (BMIs)—remain predominantly tool-like and task-oriented, with implicit short-term usage assumptions or only weak, localized coupling between living tissue and engineered components [4,5]. Consequently, biological tissues and artificial devices are frequently treated as discrete entities linked by an interface, and even advanced “substitution” systems may not fully capture the demands of chronic biointegration.

Here, we synthesize and formalize these converging developments under the term “fusion of lifeforms” as an organizing framework for a sustained, dynamic, multilevel symbiosis characterized by (i) long-term co-existence and structural/energetic compatibility, (ii) bidirectional information exchange with individualized fitting and plasticity-driven remapping, and, in stronger forms, (iii) closed-loop regulation based on endogenous feedback—together constituting cross-layer integration across structure–energy–information–cognition within a shared physiological environment. Within this framework, hybrid systems are not limited to restoring impaired functions; under appropriate conditions, they may also enable composite forms with sensing, metabolic, and cognitive capacities that extend beyond those of the original organism [6,7]. This viewpoint motivates a systematic discussion of how sustained biointegration and reciprocal signaling can reshape the distribution of sensing, regulation, and cognition between biological and artificial substrates.

Conventional human–machine interaction models face persistent limitations: restricted information throughput and closed-loop latency [8,9]; micromotion and packaging that degrade signal quality [10,11]; power and thermal constraints that limit duty cycle [12,13]; validation protocols that focus on low-level signal metrics but do not adequately capture behaviorally relevant, real-world functional gains. These bottlenecks hinder scalability from single-organ assistance to hybrid organisms endowed with stable, high-bandwidth sensing and actuation. Therefore, a broader framework is needed—one that integrates sensing, actuation, energy management, packaging, and learning algorithms into cohesive symbiotic systems with standardized metrics, in order to ensure long-term performance and safety.

The title of this review reflects an effort to consolidate several mature but fragmented research lines into a unified systems-level view. By “multi-level perception systems,” we refer to end-to-end sensing–encoding–interfacing pipelines spanning neuroprosthetics, implantable physiological sensing, sensory augmentation, and brain–computer interfaces, where signals are acquired, transformed, and ultimately translated into functional outcomes. We discuss these systems under the lens of “Fusion of Lifeforms,” emphasizing sustained structural coupling, energy cooperation, bidirectional information loops, and cognitive co-adaptation between biological and artificial components. Building on this literature, we propose a functional classification to organize representative systems, summarize recurring engineering constraints, and outline future directions toward scalable, reliable, and benchmarkable symbiotic integration.

**Figure 1:**
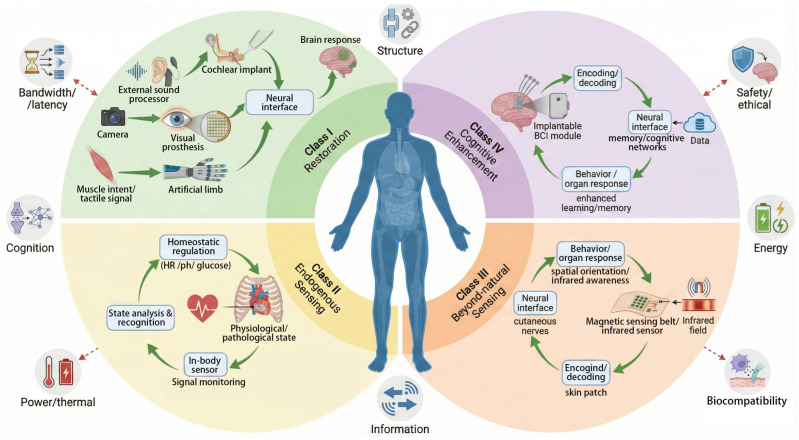
Schematic of a multidimensional enhancement framework for a “fusion of lifeform.” A semi-transparent human silhouette at the center represents the biological host. Four color-coded quadrants around the body depict four classes of enhancement systems: the upper-left Class I Restoration illustrates cochlear implants, visual prostheses, and artificial limbs with tactile feedback for reconstructing impaired sensory and motor functions; the lower-left Class II Endogenous Sensing shows implantable sensors that continuously monitor cardiovascular and other physiological/pathological states to support homeostatic regulation; the lower-right Class III Beyond-natural Sensing uses devices such as geomagnetic or infrared skin patches to convert non-natural environmental cues into signals exploitable by the nervous system; the upper-right Class IV Cognitive Enhancement features an implantable brain–computer interface that interacts with memory and cognitive networks to enhance learning and memory. Within each class, green solid arrows indicate closed-loop information flow from external or internal stimuli through sensing front ends and signal encoding/processing modules to neural or effector interfaces, ultimately generating behavioral or physiological responses. Icon sets along the outer ring represent four fundamental axes of fusion—Structure, Energy, Information, and Cognition—while red icons and red dashed arrows pointing back to the loops highlight key system constraints, including bandwidth/latency, power and thermal load, biocompatibility, and safety/ethical considerations.

In this work, we adopt the four-axis framework of “structure–energy–information– cognition” that is schematically summarized in Figure 1, and we categorize fusion of lifeform sensing systems according to their primary functional intent into four classes. Along the four quadrants in Figure 1, Class I (sensory/functional restoration) reconstructs impaired sensory and motor pathways; Class II (endogenous state sensing) continuously monitors internal physiological and pathological states, with optional therapeutic actuation; Class III (beyond-natural sensing) maps non-native environmental cues onto exploitable neural channels; and Class IV (cognitive and learning enhancement) focuses on modulating and enhancing cognition and memory through diverse neural interface and neuromodulation strategies. Section 2 defines fusion of lifeforms and their system requirements in terms of structural coupling, energy cooperation, information closed loops, and cognitive co-adaptation. Section 3 reviews the principles and recent advances of each sensing category and delineates the technological continuum from “substitution” to “fusion”. Section 4 focuses on key bottlenecks (summarized in Figure 11)—including multi-source heterogeneous integration, intra-body communication bandwidth and latency, power supply and thermal management, biocompatibility and long-term reliability, and safety and dependability in complex systems—and proposes future directions such as layered heterogeneous networks, sustainable energy provisioning, innovative biointerfaces, and system-level security frameworks. Section 5 examines ethical and regulatory issues related to self-identity, neural privacy, social fairness, and legal liability, emphasizing the need for parallel progress in technological development and institutional frameworks.

This paper classifies multi-level sensing systems in fusion of lifeforms, reviews the state of the art, and analyzes outstanding challenges. It aims to provide a systematic reference and roadmap for future research. We anticipate that, driven by interdisciplinary advances in materials, energy, communication, and intelligent algorithms, fusion of lifeforms will gradually transition from “extracorporeal assistance” to “intracorporeal symbiosis” and from “functional repair” to “capability expansion”, ultimately steering humanity toward more inclusive and extensible forms of life.

## 2. Definition and System Characteristics of Fusion of Lifeforms

Fusion of lifeforms denotes a sustained, dynamic, and bidirectional symbiosis between organic organisms and artificial systems along four operational axes—structure, energy, information, and cognition. Structurally, fusion requires chronic physical coupling and biomechanical matching in implantable, adherent, or extracorporeal configurations. Energetically, it relies on in-body/on-body power supplies, energy harvesting, and metabolically compatible power management. Informationally, it should provide bidirectional sensing–stimulation–communication, preferably implemented in closed-loop architectures. Cognitively, it leverages neural plasticity to enable co-adaptation and functional co-evolution, encompassing learning, memory, and decision support. Beyond restoring impaired function, such symbioses may give rise to composite lifeforms with perceptual, metabolic, and cognitive capacities that transcend those of the original organism.

Functionally, we classify fusion of lifeform sensing systems into four classes according to their primary functional intent within the host organism. Class I (sensory and functional restoration) comprises neuroprosthetic systems that use electrical or optical encoding with individualized calibration to recover sensory and motor function. Class II (internal-state sensing) includes in vivo monitoring platforms for otherwise imperceptible physiological states (e.g., glucose, neurotransmitters, pressure, or flow) with optional therapeutic actuation. Class III (beyond-natural sensing) refers to augmentative systems that map non-human modalities (e.g., infrared/ultraviolet, magnetic, echoic, or radiation signals) onto neural substrates through direct encoding or sensory substitution combined with adaptive training. Class IV (cognition and learning enhancement) encompasses brain–data and brain–AI interfaces that extend cognitive capacity via external information delivery and targeted neuromodulation.

### Determining the Primary Functional Intent

Many real-world systems span multiple functional intents. To make the Class I–IV labeling reproducible, we assign the *primary functional intent* using the following priority order: (1) the *stated use-case/target population* (what the system is explicitly built for), (2) the *primary endpoint* emphasized by this study (the main success metric optimized and reported), (3) the *dominant closed-loop variable* (the main variable sensed and regulated in the core loop), and (4) the *dominant design constraints* (the strongest engineering bottleneck shaping the system). When multiple intents are present, the class label refers to the primary intent; additional intents are treated as secondary and noted when relevant (e.g., “Class I, secondary Class IV”).

## 3. Functional Classification of Sensing Systems and Current Technologies

### 3.1. Sensory Restoration and Neural Fusion

The goal of restoring conventional senses is to re-establish the signal transmission pathway between external stimuli and the nervous system through deep integration of neuroprostheses with biological tissues, enabling individuals who have lost hearing, vision, touch, or smell to regain sensory function. Mechanistically, functional recovery typically depends on (i) delivering a sufficiently informative neural code that can be interpreted by downstream circuits [14,15], and (ii) leveraging neural plasticity and perceptual learning so that central pathways progressively remap and optimize the interpretation of the artificial input [16,17]. In this process, individualized fitting and, when available, closed-loop calibration act as a control layer that reduces mismatch between device encoding and the user’s neural state [18,19], stabilizes perception across contexts, and ultimately converts improved neural representations into measurable behavioral gains (e.g., speech intelligibility, navigation safety, object manipulation accuracy, and reduced cognitive load). These outcomes can be quantitatively assessed using standardized functional tests, such as the Speech Perception in Noise (SPIN) test for auditory restoration, Snellen chart for visual acuity in sight restoration, and object manipulation accuracy or sensory thresholds for tactile feedback systems. This approach allows for systematic comparison and evaluation across different systems and users (Table 1).

Unlike conventional prostheses, sensory restoration systems in fusion of lifeforms do more than substitute lost functions; they emphasize long-term in vivo coexistence and plasticity-driven functional reorganization, following a canonical pipeline of “sensing front end → feature extraction/encoding → neural stimulation or actuation → individualized calibration → behavioral validation.” Representative examples include cochlear implants [34], visual prostheses [35,36], electronic skin [37], electronic olfaction systems [38], and advanced prosthetic limbs [39,40]. Across these technologies, reproducible engineering frameworks have emerged for neural electrical/optical stimulation strategies, encoding and patient-specific fitting, wireless power and data links, and biocompatible encapsulation.

#### 3.1.1. Auditory Restoration

**Figure 2:**
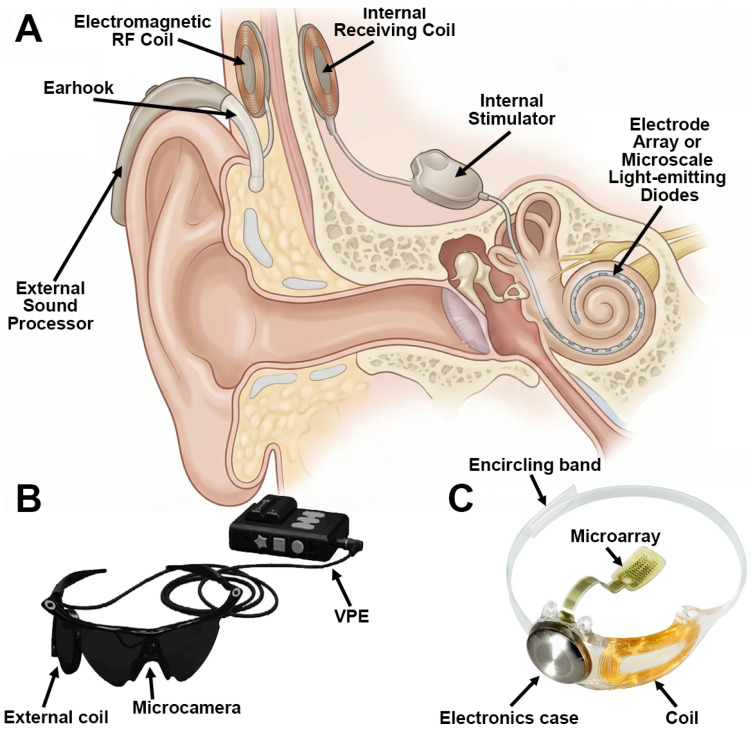
Principles of cochlear implants and components of the Argus II visual prosthesis system. (**A**) Working principles of cochlear implants based on electrical versus optical stimulation. Conventional electrical stimulation uses a multichannel electrode array to depolarize spiral ganglion neurons, but current spread often causes channel crosstalk, limiting spectral resolution. In contrast, emerging optical stimulation micro-LED arrays enables spatially confined activation, which supports more independent channels and improved sound encoding. (**B**) External components of the Argus II visual prosthesis system. The setup (Second Sight Medical Products, Inc., Sylmar, CA, USA) includes camera-mounted glasses, a video processing unit (VPU) with battery, and an external radio-frequency (RF) coil for wireless communication. (**C**) Implanted components of the Argus II system. The intraocular implant comprises a 6 × 10 epiretinal electrode array, an electronics case, and a subconjunctival RF coil. Stimulation data and power are transmitted via the RF link between the external and internal coils. (**B**,**C**) Adapted from [45], licensed under CC BY 4.0.

Cochlear implants are among the most clinically validated and widely adopted technologies in current fusion sensing systems [41]. These devices employ multichannel electrode arrays to convert acoustic signals in real time into temporally coded pulse trains that bypass damaged cochlear hair cells and directly stimulate auditory nerve fibers, thereby partially reconstructing auditory function (Figure 2A) [42]. It is estimated that more than one million individuals with severe to profound hearing loss worldwide have benefited from cochlear implantation, making it the most widely used artificial neural prosthesis to date [43,44].

In terms of encoding strategies, classical approaches such as continuous interleaved sampling and advanced combination encoder rely on band-pass filtering, envelope extraction, and channel selection to preserve critical speech information under the physical constraints of a limited number of electrodes and current spread in the cochlea [46,47,48]. However, due to insufficient effective spectral resolution and inter-channel current spread, users still experience marked limitations in noisy environments, music perception, and pitch discrimination [49,50,51].

To mitigate the well-known speech-in-noise deficit, CI research has developed dedicated front-end noise-management pipelines that complement the electrode-limited sound coding stage. Beyond classical single-microphone suppression, modern processors increasingly leverage multi-microphone directionality and beamforming to improve the effective SNR at the input of the sound coder, yielding measurable gains in challenging listening conditions [52]. In addition, spatially selective modes such as ForwardFocus further enhance target speech perception in spatially separated multi-talker interference, reporting substantial improvements in speech reception thresholds in representative test paradigms [53]. More recently, deep-learning-based speech enhancement has demonstrated improved intelligibility for CI users in noise [54], and the trend is moving toward end-to-end denoising sound coding strategies that map acoustic inputs directly to denoised electrodograms [55], potentially reducing mismatch introduced by hand-crafted front-end processing and fixed vocoders.

In recent years, research on cochlear implants has gradually shifted from merely improving basic speech intelligibility toward more intelligent and individualized paradigms. The introduction of artificial intelligence has driven substantial performance gains: deep-learning-based noise suppression and acoustic scene classification have significantly enhanced speech clarity in complex acoustic environments [21,54,56]; machine-learning-driven automated fitting, individualized coding strategies, and outcome prediction models are enabling precise adaptation to patient-specific auditory perceptual characteristics and long-term optimization during follow-up [57,58,59,60,61]. In parallel, advances in intraoperative robot-assisted electrode insertion and hearing-preservation surgical strategies have further improved implantation accuracy and protection of residual hearing [62,63,64].

Collectively, these technological developments are driving cochlear implants from early “sensory substitution” devices toward adaptive, interactive “perceptual fusion” platforms, reflecting a clear trend toward deep structural and functional integration with the nervous system.

#### 3.1.2. Visual Restoration

In the domain of artificial vision, similar fusion-oriented concepts are rapidly advancing. A variety of visual prosthesis systems—including epiretinal, subretinal, suprachoroidal retinal implants and visual cortical prostheses—aim to encode images captured by cameras or optical sensors into spatiotemporal patterns of electrical or optical/photovoltaic stimulation [23,65,66,67,68]. Through current steering and optimization of dynamic stimulation patterns, these systems seek to improve the spatial resolution and temporal continuity of phosphene perception (Figure 2B,C). Representative retinal prosthesis systems, such as Argus II, alpha IMS, and Prima, have enabled hundreds of blind patients to regain limited yet perceptible visual function [22,23,45,65,66].

In recent years, focused ultrasound stimulation has emerged as a non-invasive alternative with high spatial resolution and deep tissue penetration. For example, Lu et al. developed a two-dimensional focused ultrasound array that combines three-dimensional imaging guidance and auto-alignment technology to generate programmable ultrasonic fields, thereby achieving dynamic waveform projection at the retinal level [69]. In parallel, other researchers have proposed the use of miniature micro-LED arrays to project virtual images inside the eye for the treatment of anterior-segment blindness—a technology that can be regarded as a “miniature VR display system” embedded within the eyeball [9,70,71,72].

Beyond implantable visual prostheses, non-invasive navigation and visual substitution systems provide an important complement to visual function reconstruction. Early electronic travel aids (e.g., sonar-based canes and the NavBelt) sensed obstacles via ultrasound and delivered simple auditory cues for avoidance [73,74], but their limited feedback richness made it difficult to cope with complex urban environments. More recently, wearable and AI-enabled systems have begun to emerge. These include head-mounted virtual-vision navigation devices with integrated tactile–speech feedback and multimodal navigation–virtual companion systems [75,76]. These platforms parse camera-acquired environmental images and encode them into multipoint vibrotactile and speech feedback to guide blind users in performing real-world tasks such as independent walking and obstacle avoidance. Although the visual resolution and restorative effect of current systems remain limited, ongoing advances in optical interfaces, flexible encapsulation, biocompatibility, and AI-based image understanding and stimulation-encoding algorithms are collectively laying the groundwork for future artificial–vision fusion with higher dimensionality, stronger personalization, and greater adaptive capacity.

#### 3.1.3. Tactile Restoration

Tactile restoration aims to reintroduce touch-related cues (e.g., pressure, vibration, or shear) into the user’s somatosensory system to support object manipulation and embodiment. In practice, tactile restoration technologies can be broadly divided into non-invasive, skin-surface interfaces and invasive, subdermal interfaces. Surface approaches reconstruct a sense of touch on the skin via mechanical indentation [77], vibrotactile stimulation, or transcutaneous electrical nerve stimulation (TENS) [78]. Mechanical indentation applies localized pressure or shear to the skin through external actuators, whereas vibrotactile stimulation uses wearable vibration motors or linear resonant actuators operating at different frequencies. TENS delivers current through surface electrodes to subcutaneous nerves to evoke action potentials; however, slight deviations in stimulation intensity can readily induce pain, limiting user acceptance.

In recent years, wearable systems that integrate electromyography (EMG) decoding and mechanical indentation feedback into lightweight fabric-based prosthetic sockets have demonstrated clear advantages in providing proportional tactile feedback during prosthetic grasping [79]. Moreover, combining mechanical indentation for high spatial resolution with vibrotactile stimulation for force or intensity mapping yields superior perceived feedback quality compared with either modality alone [80]. From a receptive-field perspective, mechanoreceptors that encode skin deformation (e.g., Merkel cells and Meissner corpuscles) exhibit more localized receptive fields than vibration-sensitive Pacinian corpuscles, making mechanical indentation stimuli superior to purely vibrotactile cues in terms of spatial resolution [81].

**Figure 3:**
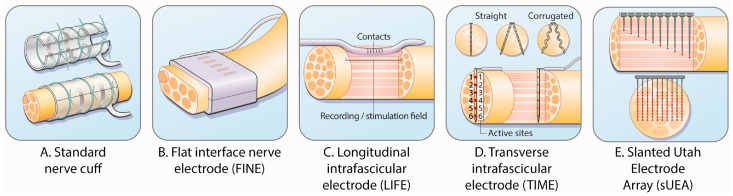
Schematics show different interface designs with the peripheral nerve: (**A**) Nerve cuff, encircling the nerve; (**B**) Flat Interface Nerve Electrode (FINE), which gently reshapes the nerve; (**C**) Longitudinal Intrafascicular Electrode (LIFE), inserted longitudinally within a fascicle; (**D**) Transverse Intrafascicular Multichannel Electrode (TIME), which penetrates the nerve transversely; and (**E**) Utah Slanted Electrode Array (USEA), providing a 3D interface with varying electrode lengths. Adapted from [87].

Subdermal approaches act directly on peripheral nerves through extraneural or intrafascicular electrodes (Figure 3 illustrates representative electrode designs), with nerve-cuff electrodes that wrap around the nerve (Figure 2A) being the most widely used [82]. These extraneural interfaces do not penetrate nerve fascicles, thereby reducing mechanical trauma and achieving biological stability over periods exceeding ten years in some cases. Nonetheless, mechanical mismatch between the electrode and neural tissue can still induce fibrotic encapsulation [83], motivating the development of all-polymer cuffs whose mechanical properties more closely match those of peripheral nerves to attenuate chronic inflammatory responses [84]. In addition, self-healing and highly stretchable conductive materials have improved the long-term robustness and reliability of prosthetic systems [85].

When combined with AI-based signal processing algorithms [86], such tactile sensor and actuator arrays can dynamically adapt to individual users’ perception thresholds and feedback preferences, progressively approximating the sensation of natural skin. It should be emphasized that tactile restoration is fundamentally a key component of closed-loop neuroprosthetic sensing systems; its system-level coordination with motor decoding and feedback encoding will be further elaborated on in Section 3.1.5.

#### 3.1.4. Olfactory and Speech Restoration

Technically, olfactory restoration typically maps chemical stimuli to neural percepts via an e-nose-based sensing and encoding front end followed by stimulation of central olfactory pathways, whereas speech restoration maps neural activity to communicative outputs (or, conversely, encodes speech features for neural delivery) through decoding/encoding algorithms coupled to appropriate neural interfaces.

Olfactory restoration is commonly built upon electronic nose (e-nose) technologies as the sensing front end. By using multichannel gas sensor arrays, feature extraction, and pattern recognition algorithms to emulate the selective responses of the olfactory epithelium, e-noses encode complex volatile organic compound profiles into “odor fingerprints.” Driven by advances in sensing materials and machine learning, such systems have recently achieved high-accuracy recognition of diverse odor classes, including disease-related breath signatures, thereby laying the groundwork for olfactory substitution and olfactory neuroprostheses [88]. On this basis, researchers have proposed conceptual “olfactory implant” architectures: an e-nose performs odor detection and feature encoding, and a miniaturized electrode array implanted in the olfactory bulb or associated olfactory pathways is driven via a wireless link to deliver electrical stimulation to central olfactory structures [27]. In principle, this approach could bypass damaged olfactory epithelium to restore olfactory function. Early human studies have shown that transnasal or olfactory-bulb stimulation can indeed evoke consciously perceived olfactory sensations, supporting the feasibility of olfactory neuroprostheses; however, challenges in spatial selectivity, long-term safety, and control of subjective odor quality remain at a very early exploratory stage [38,88,89].

By contrast, speech restoration neuroprostheses have achieved substantial clinical progress under the broader framework of brain–computer interfaces. By implanting high-density electrocorticographic grids or microelectrode arrays over or within motor and speech-related cortical areas, and coupling these recordings with deep neural networks that decode cortical activity during attempted speech, such systems can generate text or synthesize speech in real time. Recent studies in individuals with paralysis or amyotrophic lateral sclerosis (ALS) have reported decoding rates of approximately 60 words per minute with large-vocabulary sentence-level spelling accuracy, approaching or even surpassing the communication efficiency of conventional assistive communication devices [24,90,91]. Recent reviews suggest that, as high-spatiotemporal-resolution neural interfaces converge with advanced speech and language models, speech neuroprostheses may, in the medium term, support more natural continuous speech and richer emotional expression [92,93].

Overall, olfactory and speech neuroprostheses are evolving at different speeds, but both now follow a similar technical route that combines dedicated sensors, signal decoding, and targeted neural stimulation. Together, these developments are turning previously speculative forms of sensory and communicative restoration into experimentally and clinically testable interventions.

#### 3.1.5. Neuroprostheses and Perception–Action Closed Loops

**Figure 4:**
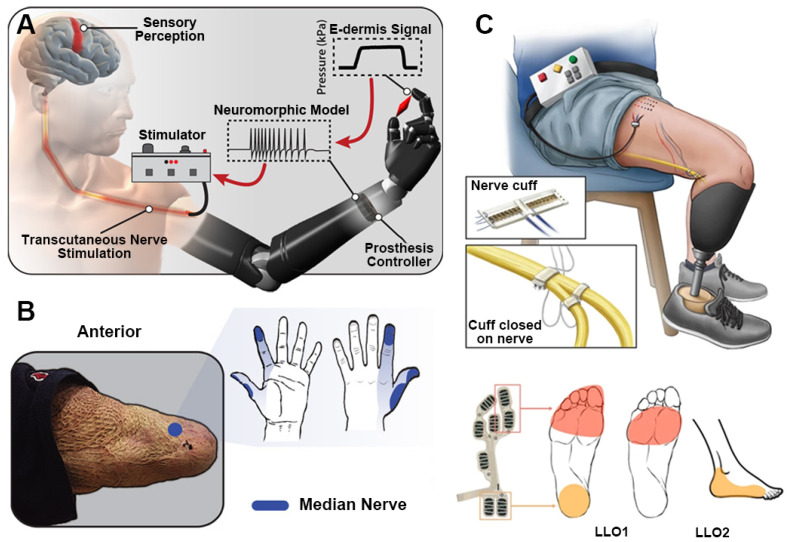
Schematic of the prosthetic sensory feedback system. (**A**) Flow of tactile feedback in a prosthetic system. During object grasping, tactile information is converted by the prosthetic controller into neuromorphic signals. These signals are delivered via transcutaneous electrical stimulation to the user’s peripheral nerves, evoking sensory perceptions such as touch and pain. (**B**) Phantom hand sensations elicited by transcutaneous electrical nerve stimulation. Electrical stimulation of the median nerve in the residual limb elicits sensations localized to the phantom thumb and index finger. (**C**) Selective stimulation using an implanted cuff electrode. A cuff electrode is implanted around the sciatic, tibial, and peroneal nerves and connected to an external stimulator. Selective stimulation of specific contacts evokes percepts (e.g., at missing toes or the heel, as reported in LL01 and LL02) that correspond to changes in plantar pressure. Adapted from: (**A**,**B**) [25]; (**C**) [103].

Technically, modern prostheses are evolving from purely mechanical devices to neurally informed systems, where decoded intent (from EMG or brain signals) drives adaptive joint/hand control, and sensorized feedback is encoded back to the user to close the human–machine loop. Modern upper-limb prostheses use EMG signals in combination with machine learning algorithms to decode user intent with high precision, transforming movement control from externally triggered commands into neurally driven, naturalistic responses [94]. Building on this, high-density EMG arrays combined with deep learning models such as convolutional and recurrent neural networks can exploit spatiotemporal muscle synergies to achieve continuous, proportional control of multiple degrees of freedom [95,96,97]. These models can also adapt in real time to user-specific contraction patterns, maintaining stable performance despite variability. Explainable AI methods help identify key EMG channels and feature dimensions, facilitating electrode layout optimization and the design of individualized training protocols, and improving system safety and debuggability [95,96]. On the output side, the integration of flexible tactile sensors, stretchable conductive hydrogels, and self-healing composite materials endows prosthetic hands with multimodal sensing of grasp force, slip, shear, and temperature [98,99,100]. Via transcutaneous or osseointegrated interfaces in combination with peripheral nerve electrodes, these signals can be encoded into position-specific “quasi-proprioceptive” and tactile feedback, significantly improving object recognition accuracy and the sense of limb embodiment [26,101]. As shown in Figure 4A,B, a novel electronic skin was developed, enabling an amputee to perceive a continuous spectrum of tactile and nociceptive sensations through the prosthesis, thereby allowing for the discrimination of object curvature and even sharpness [25]. Recently proposed high-channel-count implantable systems that combine intramuscular EMG recording with nerve stimulation further demonstrate the feasibility of implementing bidirectional neuro–electromechanical interfaces on a single implant platform, laying the groundwork for long-term in vivo symbiotic prostheses [102].

In lower-limb prosthetics, technological evolution likewise reflects a transition from “mechanical devices” toward “quasi-biological” systems. Motion control architectures that fuse EMG signals with inertial measurement units (IMUs), together with gait-phase recognition and intent-prediction algorithms, enable coordinated control of knee and ankle joints during initiation, acceleration and deceleration, and level changes such as slopes or stairs [104,105]. Microprocessor-controlled and even powered intelligent knee–ankle prostheses employ phase-dependent variable-impedance control or data-driven control policies to continuously adjust damping and output torque across different walking speeds, inclines, and uneven terrains, thereby improving terrain adaptability and gait symmetry [106,107,108,109]. More recently, deep neural networks and reinforcement learning methods have been used to learn mappings between environment conditions, gait states, and joint torques from large-scale gait datasets, allowing prostheses to maintain stable forward locomotion and obstacle negotiation even in previously unseen scenarios [109,110]. At the neural level, EEG, functional near-infrared spectroscopy (fNIRS), and hybrid BCIs are being explored for gait modulation in lower-limb prostheses and exoskeletons. By decoding movement intention or locomotor state, these systems enable “brain-controlled walking” [105,111,112]. They also offer a potential pathway for transitioning from muscle-driven to directly neural-driven control. In parallel, vibrotactile and electrical feedback systems are being developed for lower-limb prostheses to restore perception of ground contact, impact, and slope (As shown in Figure 4C). These feedback channels are designed to preserve gait stability [103] and to shorten perceptual and decision-making delays in the human–joint closed loop [113]. Across these developments, artificial intelligence is increasingly embedded in intent recognition, environment perception, control policy optimization, and feedback encoding. As a result, both upper- and lower-limb prostheses are evolving from single-function actuators into intelligent symbiotic subsystems that can co-adapt with the neuromuscular system.

Future prosthetic development is moving toward deeper integration with the nervous system. Neural interfaces—such as peripheral nerve electrodes and highly sensitive myoelectric sensors—are increasingly used to provide sensory feedback, allowing users to perceive contact forces and temperature at the prosthetic–environment interface, thereby enhancing embodiment and control precision [114,115,116]. At the same time, the combination of biomimetic materials and 3D printing has markedly improved the flexibility and individual adaptability of prosthetic structures [117], offering a new design paradigm for bio–mechanical integration. Such cross-level integration of sensing, control, and neural interfacing supports the emergence of a more continuous human–machine–neural continuum, effectively extending the functional boundaries of the body.

#### 3.1.6. Neural Coupling and Cognitive Interaction Interfaces

At the level of neural coupling and cognitive interaction, so-called telepathy-type brain–computer interfaces can be regarded as early explorations that extend traditional motor and communication BCIs toward higher-level cognitive interaction. A representative example is Neuralink’s ongoing PRIME early feasibility study, in which a neurosurgical robot implants a multichannel cortical electrode array and a fully implanted wireless module. This configuration allows individuals with high-level paralysis to control a cursor, virtual keyboard, or simple games using attempted movements or imagined actions, thereby enabling continuous interaction with computers and external devices.

Public “Telepathy” demonstrations released by the company show that several paralyzed participants can use this interface in daily life for typing, web browsing, and gaming. However, most available information comes from clinical trial registrations and company blog posts rather than from peer-reviewed reports that systematically characterize efficacy and safety [118,119,120]. In popular discourse, the term “Telepathy” is often interpreted as “direct exchange of thoughts”. In contrast, current scientific progress is more accurately described as high-bandwidth decoding of intentions and command mapping: in practice, these systems infer actions such as “select a character”, “move the cursor”, or “execute a click” from cortical activity, rather than reading out complex semantic content or abstract thoughts.

In parallel, non-invasive brain-to-brain interfaces (B2BIs) have demonstrated the feasibility of “minimal information sharing” in healthy participants. For example, BrainNet uses EEG to acquire a sender’s binary decisions and applies transcranial magnetic stimulation to deliver “yes/no” information to another participant, enabling three individuals to cooperate on a simple task [121,122,123]. These channels, however, are extremely low in bandwidth and carry very limited semantics, and they remain far from the science fiction notion of “shared consciousness”.

**Figure 5:**
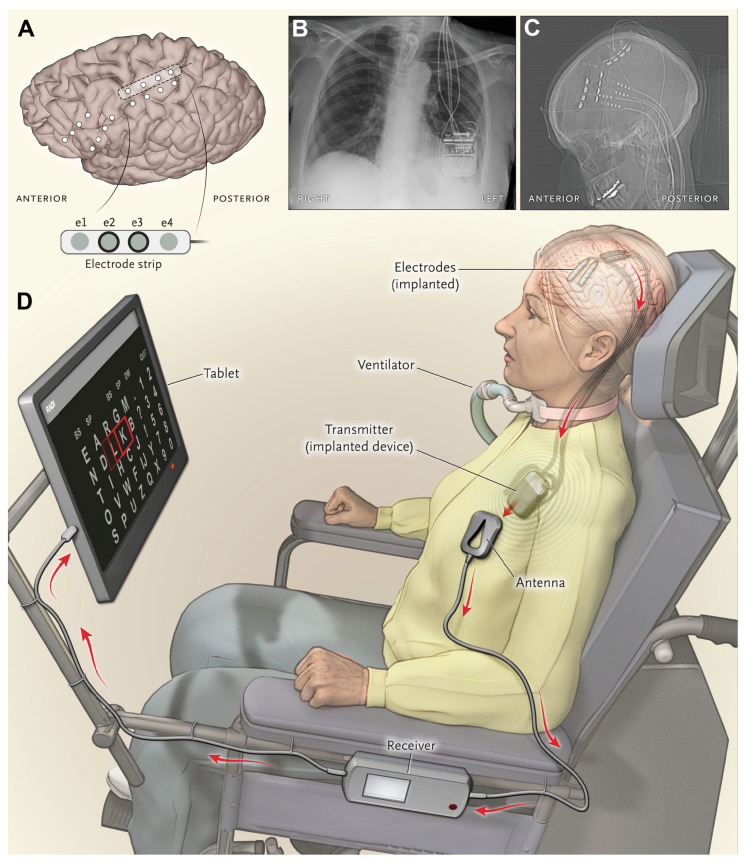
Electrode placement and system components of a brain–computer interface designed for a patient with ALS. (**A**) Electrode locations based on fusion of postoperative CT and preoperative MRI. Electrode contacts over the sensorimotor and dorsolateral prefrontal cortex are marked as white dots; electrodes e2 and e3 were selected for BCI feedback. (**B**) Postoperative chest radiograph showing the subcutaneously implanted Activa PC+S transmitter and the leads connected to the electrodes; two of the leads are shown plugged into the device. (**C**) Postoperative CT image illustrating the spatial distribution of the four electrode strips; the dot-like structures indicate the electrode connectors. (**D**) Schematic of the BCI system, including the implanted transmitter, external receiving antenna, signal receiver, and control tablet. Adapted from [124].

A more mature foundation comes from invasive communication BCIs developed for patients with amyotrophic lateral sclerosis (ALS) or complete locked-in state (CLIS), as illustrated in Figure 5. In these systems, cortical or fully implanted interfaces decode intentional selections to support spelling, sentence generation, or even continuous speech control. Such neuroprostheses provide a relatively stable channel for “intent-based communication” in individuals with severe motor impairment [124,125,126].

In summary, current neural-coupling and cognitive–interaction interfaces are best viewed as systems that decode user intentions and map them onto external commands, rather than as tools for “mind reading”. Within strict constraints on safety, power, and bandwidth, they combine high-resolution neural recording, AI-based intent recognition, and, in some cases, sensory feedback to extend the loop of attention–intention–action–feedback into digital devices or simple multi-user settings. Future extensions toward multi-brain collaboration or cognitive enhancement will depend not only on advances in large-scale neural decoding and closed-loop stimulation, but also on clear norms regarding neural privacy, agency, and emerging “neurorights”. It should be emphasized that our discussion of these systems is primarily conceptual and forward-looking, and does not constitute an endorsement of their clinical effectiveness. In particular, highly publicized demonstrations should be interpreted with particular caution by the media and non-expert audiences, and not taken as evidence of mature or widely applicable clinical therapies.

### 3.2. Endogenous Sensing and Physiological Closed-Loop Control

The primary objective of in-body sensors is to endow individuals with the ability to perceive internal vital information that is otherwise inaccessible to conscious awareness. These sensors harvest the body’s intrinsic biomechanical activities (e.g., heartbeat, respiration, gastrointestinal peristalsis) to generate potential differences in triboelectric/contact electrification interfaces, thereby providing power for sensing and wireless transmission circuits. By deploying miniaturized implantable sensing units within the body, they enable self-powered, real-time monitoring and feedback of deep physiological and pathological signals such as blood glucose, blood pressure, and electroencephalography (EEG) [127]. The functional gains of these systems can be evaluated through metrics such as the accuracy and sensitivity of physiological signal detection (e.g., ±5% accuracy for blood glucose levels, ±1 mmHg for blood pressure), the real-time responsiveness of the system (response time < 1 s), and the stability and longevity of the sensor under continuous operation (e.g., at least 1-year battery life or self-powered efficiency) (Table 1). These metrics are critical for comparing the performance of different sensing technologies in real-world clinical or daily life conditions.

Currently, in-body sensor systems are evolving from exogenous perception modalities—such as short-term monitoring and adjunct imaging—toward endogenous sensing and feedback regulation platforms that can operate stably over the long term and cooperate with the body’s physiological networks, thus realizing a paradigm shift from “external observation of life” to “intrinsic self-perception of the living system.” Ongoing research is increasingly focused on dynamic physiological monitoring and regulation [28,31,32], metabolic process recognition [128], decoding of neural electrical activity [129], and multimodal fusion, thereby laying the foundation for self-sensing medicine, human–machine symbiosis, and intelligent living systems.

#### 3.2.1. Vital Sign Sensing and Real-Time Regulation

**Figure 6:**
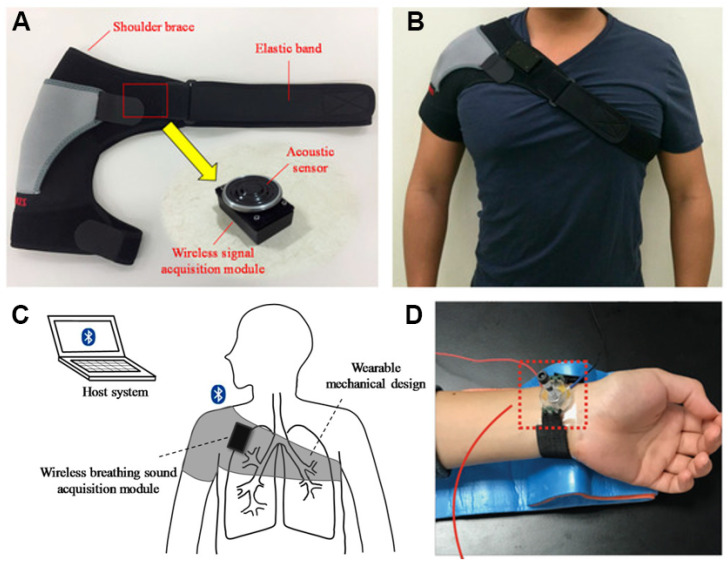
lFlexible wireless sensing system for asthma management. (**A**) Structural design of the flexible respiratory sound patch. (**B**) Schematic of on-body use, with the patch attached to the chest wall to capture breathing sounds. (**C**) System block diagram for real-time analysis of airway status. (**D**) Wrist-worn flexible blood-pressure sensor for coordinated monitoring. (**A**–**C**) Adapted from [132]; (**D**) adapted from [133].

Sensors for dynamic physiological monitoring are primarily tasked with enabling real-time, long-term tracking of vital signs such as blood pressure, respiration, and cardiac activity [130,131]. These sensors are mainly classified into wearable and implantable devices, which are tailored for continuous daily monitoring and precise perception of deep physiological states, respectively. For example, a wearable flexible respiratory sound patch attached to the chest wall acquires the spectral features of airway breath sounds to identify wheezes, thereby enabling real-time monitoring of airway narrowing and early detection of asthma [132] (Figure 6A–C). Wearable blood-pressure sensors detect capacitance changes induced by radial arterial pulsation to obtain pulse pressure waveforms, which are subsequently converted into blood pressure parameters, thus achieving beat-to-beat continuous blood pressure monitoring [133] (Figure 6D). However, such wearable devices still mainly rely on body-surface signals and are susceptible to attenuation through skin conduction and motion artifacts, making it challenging to realize continuous, high-precision monitoring of deeper physiological processes.

To achieve a genuinely fusion of lifeforms system, research is progressively shifting toward implantable platforms. Implantable physiological sensors acquire in situ physiological signals through direct contact with tissues and support closed-loop regulation. In the cardiac system, flexible self-powered sensing and regulation architectures based on triboelectric nanogenerators (TENGs) establish a technological pathway from “cardiac activity monitoring → refined cardiac function identification → myocardial functional intervention and repair.” Early miniature implantable subendocardial pressure sensors (SEPS) employed TENGs to realize in situ, self-powered detection of intracardiac pressure and arrhythmias [28]. The subsequently upgraded gapless Nano-Structured Triboelectric Nanogenerator (NSTENG), which eliminates mechanical spacing, achieves higher sensitivity and enables real-time recording of complete pulse waveforms and subtle myocardial motions [29]. On this basis, the self-generated TENG signals can also be directly used to electrically stimulate cardiomyocytes, promoting cardiomyocyte maturation and functional restoration of myocardial tissue, thereby extending the role of the system from “monitoring” to “therapy” [30].

Beyond the cardiac system, sensors for dynamic physiological monitoring have also demonstrated remarkable potential in other organ systems. In the gastrointestinal tract, Yao et al. developed an implantable self-powered vagus nerve stimulation device [31], in which a nanogenerator is attached to the gastric wall to harvest peristalsis-induced mechanical deformation and generate biphasic electrical pulses that stimulate the vagus nerve to modulate appetite, thereby achieving effective body-weight control. In the urinary system, Hassani et al. integrated a triboelectric sensing module with a shape-memory alloy actuator [32]; the former continuously senses bladder wall tension to determine the filling state and uses this as a trigger signal, while the latter actively compresses the bladder to induce voiding, thus constituting an implantable closed-loop control system capable of self-detection and autonomous urination. In orthopedics, a TENG-based self-powered implantable electrical stimulator upregulates intracellular ${\mathrm{Ca}}^{2+}$ signaling in osteoblasts, promoting cell proliferation and bone matrix formation, thereby providing a long-term therapeutic strategy for osteoporosis and related fractures [33] and contributing to a gradually integrated technological framework that spans from monitoring to therapy [134].

Overall, sensors for dynamic physiological monitoring are evolving from body-surface perception toward in situ tissue sensing and, in the implantable domain, are leveraging self-powered mechanisms such as TENGs to achieve a transition from passive monitoring to closed loop, actively interventional operation. Existing studies in the cardiac, gastrointestinal, urinary, and skeletal systems have demonstrated their potential for long-term stable power supply, high-fidelity acquisition of deep physiological signals, and precise regulation based on physiological feedback, thereby outlining an integrated in vivo pathway that spans “monitoring–identification–regulation–repair.”

#### 3.2.2. Metabolic Process Sensing and Chemical Homeostasis

Sensors for metabolic process recognition infer the body’s metabolic state by tracking changes in key chemical constituents in biofluids or tissues, among which glucose, pH, and dopamine are typical indicators. Glucose-monitoring technologies have evolved from finger-stick blood sampling to continuous glucose monitoring (CGM) and, more recently, to wearable non-invasive sensors. Conventional finger-stick methods rely on disposable test strips and colorimetric analysis, which only provide discrete glucose readings and fail to capture dynamic fluctuations. CGM systems, in contrast, implant electrochemical sensors into the subcutaneous tissue to continuously detect glucose concentrations in interstitial fluid and wirelessly transmit data, thereby yielding a dynamic glucose profile [129,135]. Commercial CGM devices based on this principle, such as Dexcom G7 and FreeStyle Libre 3, have achieved significant improvements in wear duration, measurement accuracy, and connectivity stability [136]. Recent research has shifted toward non-invasive wearable sensors that analyze sweat or interstitial fluid, utilizing enzymatic or non-enzymatic electrochemical reactions to achieve continuous, needle-free monitoring and thereby improve comfort and user compliance [137]. For instance, recent advances in flexible microfluidic platforms have enabled multiplexed, real-time monitoring of sweat metabolites and electrolytes, such as uric acid, pH, and K^+^, with high sensitivity and mechanical robustness during physical activity [138]. Although some studies suggest that glucose monitoring will ultimately move toward fully non-invasive approaches, such devices currently face challenges in signal calibration and long-term stability and are unlikely to completely replace CGM in the near term. Consequently, CGM systems and non-invasive wearable sensors should be viewed as two parallel developmental pathways: the former continues to advance in accuracy and intelligent data analytics, whereas the latter focuses on enhanced comfort and widespread accessibility [136].

Beyond blood glucose, implantable nanostructured pH sensors employ porous silicon or polymeric interfaces that are sensitive to changes in hydrogen-ion concentration to enable in situ, continuous monitoring of tissue microenvironment pH, thereby supporting the assessment of inflammatory progression, wound healing, and tumor microenvironment remodeling [128]. In addition, microelectrode-based electrochemical sensing technologies can record dopamine redox signals directly in the brain, enabling continuous dynamic monitoring of dopamine levels and providing a basis for endogenous closed-loop stimulation or medication adjustment according to neural state in patients with Parkinson’s disease, depression, and related disorders, with the goal of maintaining stable motor and affective function [139]. These advances are driving real-time perception of human metabolic status and the development of personalized medicine.

Overall, sensors for metabolic process recognition are shifting from external monitoring toward an in vivo cooperative sensing paradigm characterized by in situ operation and long-term coexistence with tissues. By continuously and dynamically tracking metabolic indicators such as blood glucose, pH, and dopamine, these systems can provide real-time feedback and adaptive regulation in response to changes in the internal physiological environment. In this way, sensors are no longer merely external auxiliary devices, but become integrated physiological units that co-participate in the maintenance of bodily homeostasis, embodying a broader trend toward human–device integration.

#### 3.2.3. Neural Signal Decoding and Interaction Interfaces

**Figure 7:**
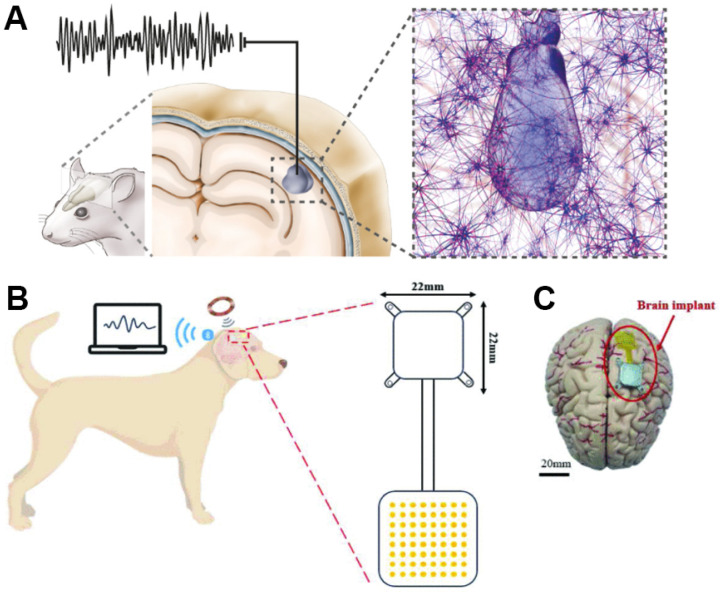
Implantable neural sensing interfaces for high-fidelity brain-state monitoring. (**A**) Schematic of a high-signal-to-noise-ratio conductive hydrogel suitable for acute and chronic inter-cortical and epidural neural recordings, illustrating its interaction with neurons in the mouse brain. (**B**) Schematic and enlarged view of a wireless miniature brain–computer interface system implanted in a Labrador retriever. (**C**) Conceptual illustration of the implantation site of the wireless miniature brain–computer interface system in the human brain. (**A**) Adapted from [141]; (**B**,**C**) adapted from [144].

Sensors for interpreting neural electrical activity record and analyze endogenous brain electrical signals to enable real-time perception of brain functional states and neuromodulatory processes. In contrast to communication-oriented brain–computer interfaces that primarily decode motor intentions (see Section 3.1.6), these systems emphasize “endogenous state sensing,” that is, identifying internal brain states—such as epileptic seizures, levels of consciousness, sleep rhythms, and emotional fluctuations—from neural signals and converting them into observable external readouts or controllable parameters. Implantable brain–computer interfaces (iBCIs) are the core technological paradigm in this field: by implanting high-density microelectrode arrays into the central nervous system to record neuronal spiking activity and decoding it algorithmically, they establish an internal sensing pathway for brain states within the body [140]. To support long-term, stable monitoring of endogenous signals, advances in interface materials and system integration are crucial. Flexible, conductive hydrogel electrodes can match the mechanical modulus of brain tissue, markedly reducing post-implantation inflammatory responses and enhancing signal stability [141,142] (Figure 7A). Nanomaterial-modified electrode interfaces further improve the spatial resolution and signal-to-noise ratio of neural recordings [143]. On the system integration level, organizations such as Neuralink have developed high-throughput, fully implantable recording devices that employ surgical robots to precisely insert kilo-channel flexible electrode threads, enabling parallel wireless acquisition of brain activity across multiple regions. Other studies have demonstrated a 64-channel miniature flexible electrode array encapsulated in a 22 × 22 mm^2^ titanium housing, achieving wireless power transfer and long-term in vivo signal acquisition [144,145] (Figure 7B,C). Collectively, these advances are driving neural interfaces to evolve from external, add-on recording devices into in situ implantable sensing units that can coexist with neural tissue over the long term, continuously capture deep neural state information, and support closed-loop feedback.

Leveraging high-quality neural signal acquisition and intelligent decoding algorithms, neural interfaces are increasingly being used to construct dynamic models of an individual’s internal physiological state and to implement adaptive closed-loop regulation. For example, in epilepsy therapy, responsive neurostimulation systems detect abnormal epileptiform discharge patterns and promptly deliver electrical stimulation to interrupt pathological activity at an early stage of seizure development [146]. Compared with conventional continuous stimulation, such closed-loop strategies significantly reduce seizure frequency while minimizing unnecessary stimulation-related side effects [147]. In the context of consciousness and sleep monitoring, EEG-based anesthesia depth indices have been introduced to assess and modulate the level of consciousness during anesthesia, thereby reducing the risk of intraoperative awareness [148,149]. Emerging work has also begun to decode emotional and cognitive states: implantable devices that monitor activity in specific brain regions can identify neural network signatures associated with conditions such as depression and, when needed, deliver brain stimulation or pharmacological interventions to stabilize patients’ mood [139]. In Parkinson’s disease, pathological β-band oscillations recorded via implanted electrodes serve as biomarkers to drive adaptive deep brain stimulation (DBS), enabling individualized closed-loop control of motor symptoms while improving therapeutic efficacy and reducing stimulation power consumption [150,151]. By integrating in vivo sensing with therapeutic intervention, neural-signal-decoding interfaces are thus transitioning from purely passive monitoring toward active regulation.

Taken together, neural-signal-decoding and interaction interfaces play a unique role within the fusion of lifeforms-system framework—rather than serving to read out thoughts for controlling external devices, they function as internal “sense organs” and “regulators” of the body, continuously tracking fluctuations in intrinsic brain states and providing adaptive feedback control. With the convergent development of implantable/non-invasive neural sensors and artificial intelligence algorithms, such interfaces are poised to become key components for maintaining neural homeostasis, forecasting pathological events, and delivering personalized interventions, thereby achieving deep integration of human–machine systems at both structural and functional levels.

#### 3.2.4. Multimodal Sensing and System Integration

**Figure 8:**
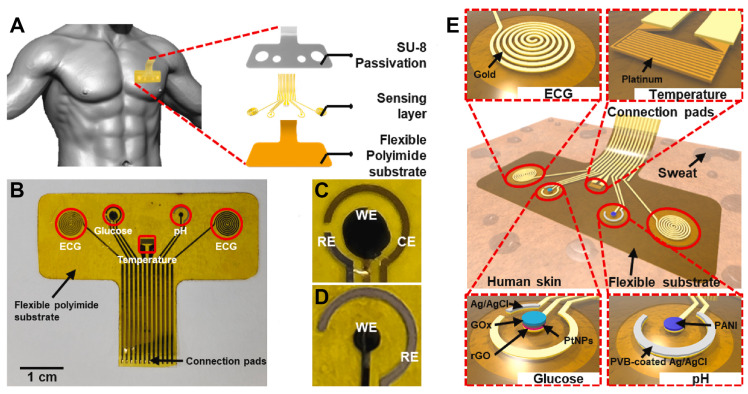
A wearable hybrid skin patch for continuous physiological monitoring. The figure presents (**A**) an exploded-view schematic of the patch assembly, (**B**) an optical image of the final device, and detailed views of its key components, including (**C**) the glucose and (**D**) pH sensors, culminating in (**E**) a magnified perspective of the integrated sensing platform. Adapted from [153].

Multi-sensor networks refer to configurations in which wearable and implantable sensors are wirelessly interconnected to form an Internet of Bodies (IoB) or wearable–implantable body sensor networks (WIBSNs), thereby enabling multimodal cooperative sensing of vital signs, biochemical markers, and physiological signals [152]. Within wearable devices, substantial progress has been made in the high-level integration of multiple sensing units. For example, Yoon et al. integrated sweat-glucose (Figure 8C), potentiometric pH (Figure 8D), temperature, and dry-electrode electrocardiogram (ECG, Figure 8B) sensors into a single flexible skin patch (Figure 8A), enabling multimodal, synchronous monitoring of metabolic status and cardiac electrophysiological activity, and supporting real-time interaction with bodily signals for continuous surveillance and dynamic management of chronic metabolic diseases [153]. Ma et al. developed a smart contact lens that employs enzymatic electrochemical biosensing electrodes to read metabolite concentrations in tears and pressure-/capacitance-based structures to sense corneal deformation associated with intraocular pressure, thereby achieving non-invasive, real-time monitoring of glucose, lactate, and intraocular pressure and providing a continuous-monitoring modality for metabolic disorders [154].

Building on these developments, multi-sensor networks are further being extended to cross-layer cooperation between wearable and implantable devices, which are wirelessly interconnected to form an integrated body-sensing network. For instance, implantable sensors that monitor glucose, pH, or neural electrical activity in vivo can be linked via data connections to external wearable devices, enabling information integration across tissue layers [155]. In such architectures, in-body sensors are responsible for in situ acquisition of deep physiological signals, on-body devices serve as relays and provide auxiliary monitoring, and mobile terminals and cloud platforms execute analysis and feedback. Together, they establish a closed-loop pathway of “in-body sensing—on-body interaction—cloud-based decision-making,” enabling continuous cross-tissue information fusion and dynamic regulation.

Taken together, in-body sensors are evolving from localized signal acquisition devices into active sensing and regulation systems that can reside in the body over the long term and operate in coordination with physiological functions. Physiological dynamic-monitoring modules enable deep, time-resolved perception that extends from the body surface to in situ tissue sensing; metabolic process recognition modules provide continuous tracking of internal chemical homeostasis; neural electrical activity decoding modules achieve real-time reading and writing of neural circuit states; and multi-sensor networks further link in-body and on-body devices, as well as local and systemic levels, into a unified information-circulation pathway. In this way, sensors are evolving from externally attached tools into integrated functional units within the organism. These units actively participate in maintaining physiological homeostasis, enabling cooperative regulation and internal feedback control. This evolution signifies a paradigm shift from “devices serving the organism” to “devices becoming constituent parts of the organism,” thereby advancing the development of true fusion of lifeforms.

### 3.3. Suprasensory Augmentation and Channel Mapping

This class of technologies aims to introduce new senses that extend beyond the native human repertoire. Sensory dimensions that are normally inaccessible to humans—such as infrared and ultraviolet radiation, geomagnetic and electromagnetic fields, and ultrasound—are mapped onto existing tactile, auditory, or visual channels (Table 2). Through training that exploits neural plasticity, users can form stable perceptual representations and achieve quantifiable task benefits.

A representative example is geomagnetic sensing. The feelSpace vibrotactile belt uses a ring-shaped array of tactors around the waist to continuously indicate magnetic north [156]. After up to 15 months of training in natural environments with 9 participants, users developed a stable “sense of north” and showed significant improvements in orientation and navigation tasks. This work directly demonstrates that sensorimotor contingencies can be learned and transferred [165,166].

Infrared thermography, which is already widely used in night vision and medical thermal imaging, has also been formalized as a new sensory source [167]. In animal models, researchers have mapped infrared intensity onto electrical stimulation of the primary somatosensory cortex (S1) at different frequencies [157]. Rats learned to detect infrared cues, and the new modality coexisted with native touch rather than replacing it, effectively creating an additional sensory channel. Clinically, for users of visual prostheses such as Argus II, thermal cameras have been integrated into the visual pipeline [158]. The infrared input is semantically simplified and fused with the existing visual stream. This approach improves night-time navigation, human-body detection, and environmental awareness. A key principle is to replace high-redundancy video streams with low-bandwidth, high-value infrared features, thereby improving the signal-to-noise ratio at the source and reducing the decoding burden on the cortex.

Overall, suprasensory systems extend human and animal capabilities beyond their natural sensory range, allowing users to exploit otherwise inaccessible cues for navigation and interaction with the environment. To make these “extra” senses reliable in daily use, the whole chain—from sensing-source selection and feature compression to channel mapping and training—must be designed as an integrated system that yields stable, behaviorally meaningful improvements.

### 3.4. Cognitive Enhancement and Intelligent Integration

As a higher-level manifestation of fusion of lifeforms, cognitive enhancement aims to overcome the biological brain’s intrinsic limitations through deep integration of neural interfaces with external intelligence, thereby achieving generational leaps in higher-order cognitive functions such as learning, memory, and decision-making. This endeavor centers on constructing a bidirectional closed-loop cognitive coupling system: internal neural activity is continuously sensed and decoded in real time, while targeted stimulation or cloud-based intelligent feedback is delivered to the brain, optimizing neural plasticity and information-processing efficiency [168]. The field has evolved from rudimentary brain-state decoding toward advanced fusion pathways, including cognitive capacity enhancement, memory modulation, cloud-based knowledge collaboration, and multi-brain cooperation (Table 2). Key metrics to evaluate the functional gains of these systems include improvements in memory recall (e.g., Rey AVLT, Digit Span test), decision-making efficiency (e.g., Iowa Gambling Task), and learning speed (e.g., learning task completion time). Furthermore, neural plasticity can be assessed through changes in brain activity, measured via EEG or fMRI, in response to real-time feedback and closed-loop stimulation. Together, these technologies are shifting cognitive augmentation from a purely biological paradigm to a symbiotic human–machine integration paradigm, endowing fusion of lifeforms with a core engine of intelligence.

#### 3.4.1. Cognitive Enhancement and Symbiotic Regulation

To achieve deep integration of the “fusion of lifeforms” at the cognitive level, BCI technologies are steering the transition from traditional external auxiliary stimulation toward a new paradigm of long-term symbiotic regulation with the brain. By constructing closed-loop systems that couple neural signal decoding with real-time feedback, this approach enables individuals to learn to modulate their own brain activity, thereby enhancing attention, emotional regulation, and memory, and ultimately achieving active intervention in cognitive function [168]. Studies have shown that physical neuromodulation techniques such as transcranial magnetic stimulation, deep brain stimulation, and focused ultrasound can improve cognitive function and alleviate neurological symptoms by modulating neural circuits involved in attention, memory, and decision-making, leveraging mechanisms of neural plasticity [169]. In particular, flexible neuromorphic electronics supporting near-sensor and in-sensor computing paradigms offer bio-inspired data compression and parallel processing, paving the way for seamless cognitive fusion in wearable and implantable platforms [170].

Building on these mechanisms, related technologies have been extended to multiple application scenarios closely linked to functional restoration of the organism. Tsai et al., for example, conducted closed-loop neurofeedback training in older adults, during which participants received real-time EEG feedback while performing tasks; the results showed significant improvements in attention, working memory, and executive control, indicating that feedback-based regulation can directly enhance cognitive performance. On this basis, Peterson et al. introduced a co-adaptive decoding framework in motor imagery tasks. When the system dynamically updated its parameters according to the subject’s neuromodulation performance, increased inter-class separability of neural representations and improved decoding accuracy were observed, demonstrating that closed-loop regulation can drive the brain to proactively reorganize its own activity patterns [160]. In addition, Matt et al. applied transcranial pulsed ultrasound to patients with Alzheimer’s disease; the intervention group exhibited significantly higher scores on cognitive scales than the control group and showed enhanced activation of attention–memory networks on functional MRI. These findings provide circuit-level evidence of plasticity and indicate that the aforementioned representational reshaping can be consolidated at the network level [161].

Taken together, the technological trajectory of cognitive enhancement is shifting from short-term exogenous stimulation or behavioral compensation toward a long-term symbiotic regulation mechanism jointly driven by neural learning and circuit plasticity. Within this mechanism, the brain forms new neural representations through feedback, while external systems consolidate these representations via circuit-level modulation so that the maintenance of cognitive function no longer depends on transient interventions but is jointly sustained and continuously reshaped by both human and machine. As a result, cognitive processes progressively transition from passive responses to open, co-regulated dynamics, laying the foundation for the long-term stable operation of the “fusion of lifeforms” at the cognitive level.

#### 3.4.2. Memory Enhancement and Precision Intervention

At the level of memory enhancement and precision intervention, implantable brain–computer interfaces and deep brain stimulation are shifting from mere symptomatic relief toward “co-writing” memory traces within critical windows of memory formation. Current studies have shown that it is possible to record activity patterns in the hippocampus–medial temporal lobe and associated neocortical regions while individuals perform episodic memory tasks, use machine learning models to distinguish “high-memory” from “low-memory” states, and trigger closed-loop stimulation only when encoding falls into a low-efficiency state, thereby significantly improving subsequent recall performance [162,171]. In primate and human work on “hippocampal cognitive prostheses,” researchers have employed multi-input–multi-output (MIMO) models to reconstruct ensemble firing patterns along the CA3 → CA1 pathway and then replay the predicted encoding trajectories via electrical stimulation, partially restoring or even enhancing working memory and delayed matching performance under conditions of hippocampal damage or task interference [163,172].

Going a step further, closed-loop stimulation systems based on phase-locking algorithms can track theta oscillations in hippocampal–cortical networks in real time and deliver brief stimulation at the phase of maximal excitability. Experiments demonstrate that such phase-precise interventions improve memory performance more effectively and with fewer side effects than continuous open loop stimulation [164,173]. Recent reviews and quantitative analyses also indicate that memory-enhancing BCIs are moving from simple continuous stimulation toward adaptive closed-loop control triggered by electrophysiological biomarkers: on the one hand, high-density recording and modeling are used to capture individualized memory-encoding dynamics; on the other hand, minimal-dose stimulation is applied within appropriate spatiotemporal windows to reshape plasticity, thereby directly boosting the formation and retrieval of declarative memory without relying on long-term behavioral training. From the perspective of a fusion of lifeform, these approaches effectively outsource part of the “memory-writing” process to neural interfaces and algorithms. This transformation turns memory from a purely endogenous function into a human–machine co-executed physiological process, providing an experimentally verifiable pathway for precise interventions in both memory-disorder treatment and cognitive enhancement for healthy individuals [174].

#### 3.4.3. Cloud Intelligence and Cognitive Extension

In the realm of “cloud intelligence and cognitive extension,” brain–cloud cooperation is regarded as a key pathway for embedding the individual brain into a distributed intelligent system. The basic concept is as follows: brain signals are acquired via wearable EEG or implantable BCIs, undergo preliminary preprocessing and encryption at local edge nodes, and are then uploaded to the cloud, where large-scale AI models perform pattern recognition and state estimation. The decoded results or optimized control/stimulation parameters are subsequently transmitted back downstream and fed into the cognitive process via neural stimulation or environmental feedback. In this way, a closed-loop cooperative architecture of “human brain–edge computing–cloud intelligence” is established [175].

**Figure 9:**
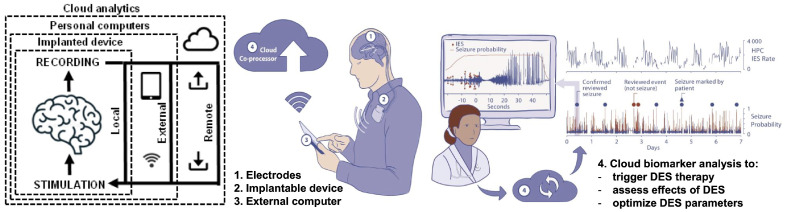
Closed-loop framework for adaptive direct brain stimulation (adaptive direct electrical stimulation). The left panel illustrates potential scenarios for triggering responsive stimulation across three progressively more distributed and externalized brain–machine interface configurations. The right panel presents an extended example of a data-processing loop, in which neural signals flow from the implantable device to external computers and the cloud for collaborative analysis. By performing real-time or delayed biomarker analysis on continuously recorded intracranial electroencephalography(iEEG) signals (e.g., using machine-learning methods to detect epileptic activity), the system determines the timing and parameters of stimulation, thereby enabling state-dependent optimization of therapeutic and cognitive functions. Adapted from [177].

For example, Rizzo et al. designed a cloud-based brain–computer interface system driven by steady-state visual evoked potentials (SSVEP) [176]. Using wearable EEG devices in combination with an embedded edge platform (Raspberry Pi 4), they achieved real-time interactive control of a cognitive building environment and demonstrated that support vector machines and random forest algorithms can effectively perform SSVEP classification on the edge device, with accuracies exceeding 97%. At a deeper neuromodulation level, the fields of memory enhancement and deep brain stimulation have proposed the concept of a “brain co-processor”: through wireless bidirectional communication, implantable recording/stimulation devices are integrated with smartphones and cloud computing, allowing large-scale neural data to be continuously analyzed in the cloud and stimulation strategies to be adaptively adjusted based on electrophysiological biomarkers, thereby enabling long-term, fine-grained regulation of memory and cognitive functions [173,177] (as shown in Figure 9).

At the theoretical level, the “human brain/cloud interface” proposed by Martins and colleagues sketches a visionary blueprint for tightly coupling the human brain with cloud-based artificial general intelligence (AGI) systems. It emphasizes that, by integrating ultra-high-bandwidth neural interfaces with cloud computing resources, it may eventually become possible to realize cross-individual knowledge sharing and amplification of collective intelligence while simultaneously highlighting profound risks related to neural privacy, security, and personal agency [178].

Overall, cloud intelligence and cognitive extension provide fusion of lifeforms with a technical pathway akin to an “external intelligent cortex.” This shifts cognitive enhancement beyond local brain region stimulation toward a continuously co-regulated cognitive ecosystem, shared between humans and distributed cloud intelligence.

#### 3.4.4. Inter-Brain Collaboration and Collective Intelligence

As a key pathway for realizing multi-brain collaborative sensing and cognition within the “fusion of lifeforms,” brain-to-brain interfaces (BBIs) combine BCIs with computer–brain interfaces (CBIs) to establish direct information-transfer channels between brains. In doing so, they bypass the constraints of conventional language and motor behavior and enable collaboration at the neural level. In recent years, BBI technologies have rapidly evolved from early unidirectional animal experiments into comprehensive systems encompassing non-invasive human–human communication, cross-species bidirectional control, and high-precision neuromodulation [179].

**Figure 10:**
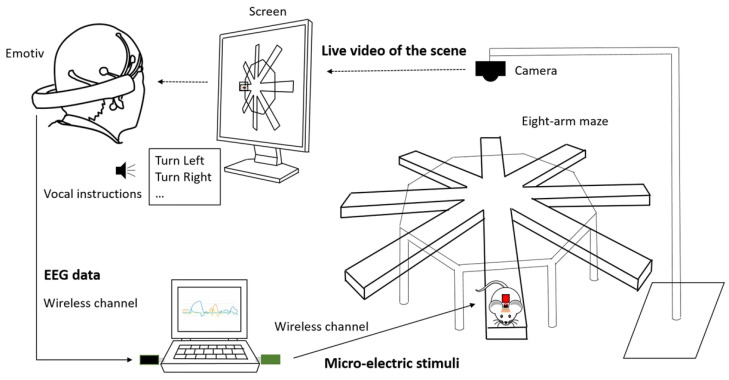
Overall experimental architecture of the wireless BBI system. On the left, an EEG-based BCI decodes the human user’s left- and right-hand motor imagery and eye-blink commands, which are mapped onto three navigation commands: left turn, right turn, and forward. On the right, a wireless microstimulation module delivers these commands in real time to electrodes implanted in the rat brain, with stimulation sites in the SIBF region (turning behavior) and the medial forebrain bundle (MFB, forward locomotion/virtual reward), enabling human intention to directly drive continuous walking and navigation in the rat. Adapted from [182].

In the domain of non-invasive human brain communication, systems that integrate EEG with transcranial magnetic stimulation (TMS) have demonstrated the transmission of information at the level of conscious awareness between individuals. Grau and colleagues decoded a sender’s motor intention using EEG and induced phosphenes in a receiver’s visual cortex via TMS, achieving the first Internet-mediated brain-to-brain communication [180]. Building on a similar architecture, Jiang and co-workers developed “BrainNet,” which enables multiple participants to engage in collaborative decision-making via inter-brain information exchange, marking a new stage of multi-brain cooperation [121]. In the direction of cross-species bidirectional control, Yoo [181] and Zhang [182] independently demonstrated real-time human control of rat tail movements and maze navigation, respectively, with intracortical microstimulation (ICMS) delivering somatosensory feedback from the animal back to the human operator, as schematically illustrated in Figure 10. Together, these studies provide an initial bidirectional “perception–action” closed loop across species and lay the groundwork for cross-species fused perception. In parallel, advances in high-precision neuromodulation and emerging brain network technologies are substantially enhancing BBI performance: Lee and colleagues replaced TMS with focused ultrasound (FUS) to achieve more spatially precise somatosensory activation of specific brain regions [122], whereas Lu and co-workers used optogenetics combined with optical fiber recording to construct an “optical BBI” between mice, enabling ultra–high-speed transmission of motor information—two to three orders of magnitude faster than conventional electrophysiological approaches—and greatly expanding information throughput and response speed [183].

Overall, BBI technologies are progressing along three major axes: increasing non-invasiveness, cross-species integration, and high-precision, high-speed operation. This trajectory is gradually blurring the boundaries between individual brains and provides crucial technical support for constructing fusion of lifeforms in which perception and cognitive resources are deeply interconnected and shared at the neural level. At the same time, integrating heterogeneous systems while ensuring stable, safe, and efficient multi-layered sensory fusion remains a central challenge for future research.

Collectively, the various technological pathways for cognitive enhancement—from neuromodulation and memory intervention to cloud-based integration and inter-brain collaboration—are reshaping the cognitive architecture of fusion of lifeforms at multiple levels. Through real-time interactive sensing–regulation closed loops, these approaches not only optimize higher-order brain functions such as attention and working memory but also embed individuals within distributed intelligent networks, enabling on-demand allocation and leapfrog expansion of cognitive resources. This progression marks the transition of cognition from a closed, purely intracranial process to an open system defined by deep cooperative coupling between neural and machine intelligences, thereby establishing the cognitive-level foundation of fusion of lifeforms.

## 4. Interface Integration, System-Level Challenges, and Future Directions

### 4.1. Technical Challenges and Bottlenecks

#### 4.1.1. Challenge 1: Complexity of Multi-Source Heterogeneous Sensing Fusion

**Figure 11:**
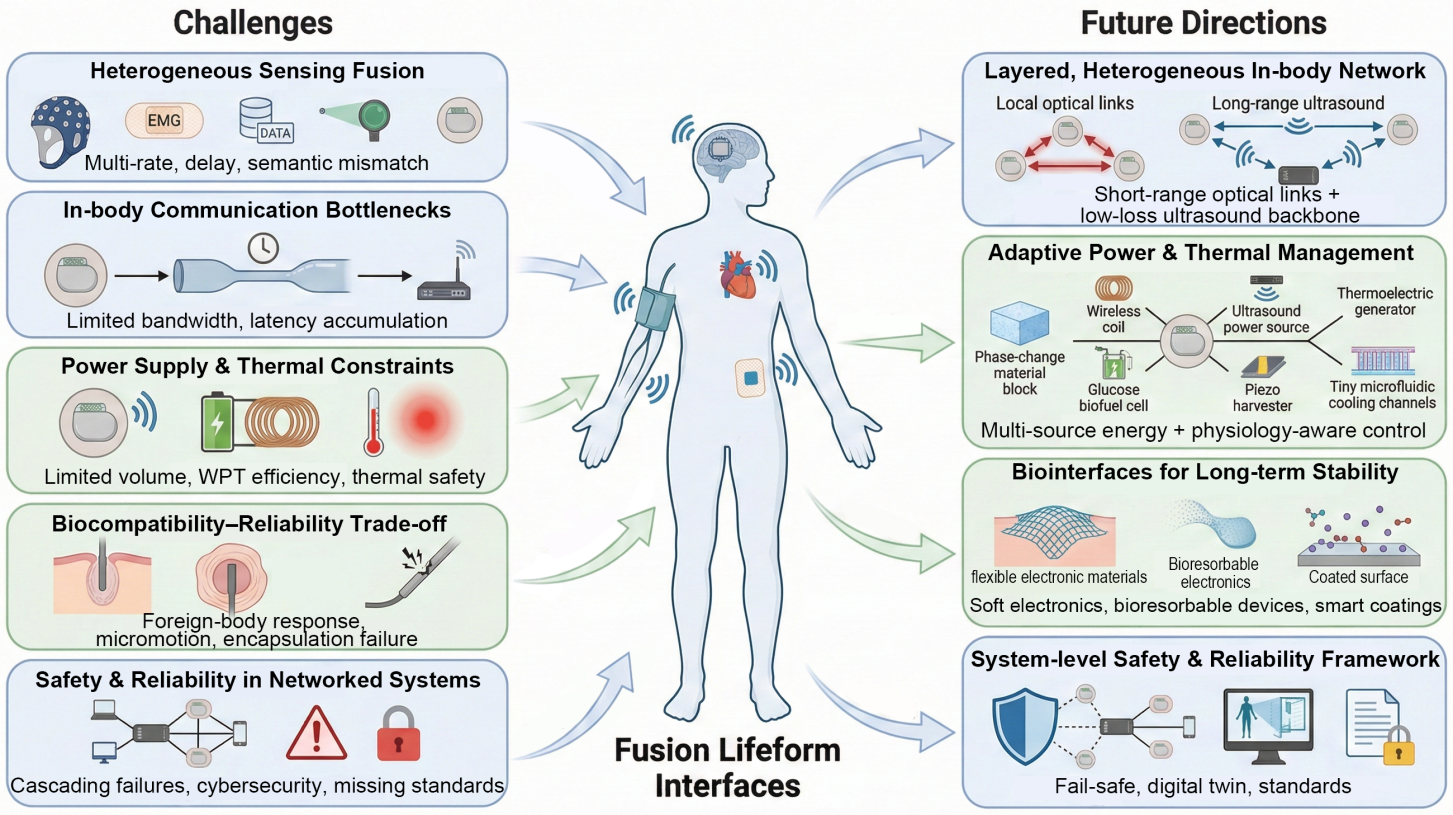
System-level challenges and future directions for fusion of lifeform interfaces. The left panel summarizes five key bottlenecks in building multi-implant, closed-loop fusion interface systems: (1) multi-source heterogeneous sensing fusion across modalities with mismatched sampling rates, delays, and semantic incompatibilities; (2) in-body communication bottlenecks due to limited channels/bandwidth and latency accumulation; (3) stringent power-supply and thermal constraints arising from limited implant volume, wireless power-transfer efficiency, and tissue thermal safety; (4) the biocompatibility–reliability trade-off driven by foreign-body response, micromotion, and long-term encapsulation degradation; and (5) safety and reliability risks in networked systems, including interference, cascading failures, cybersecurity threats, and the lack of standardized benchmarks. The right panel highlights four corresponding directions to address these challenges: layered heterogeneous in-body networks (short-range NIR optical clusters coupled to an ultrasound backbone), sustainable and physiology-aware power/thermal management using multi-source energy and adaptive cooling, advanced biointerfaces enabling long-term stability (soft electronics, bioresorbable devices, and smart coatings), and system-level safety frameworks incorporating redundancy, anomaly detection, fail-safe operation, cybersecurity, standardization, and digital-twin–assisted validation.

Fusion of lifeform systems typically include multiple heterogeneous sensing and stimulation devices distributed inside and outside the body (as shown in Figure 11). Coordinating their timing and data fusion is inherently difficult. Sensing nodes at different locations often operate at different sampling rates and experience different transmission delays. Precise synchronization is required to maintain the stability of closed-loop control [184]. Calibration is also complex. Each sensor must be tuned to the individual’s physiology, while long-term implantation leads to device aging, signal drift, and scar-tissue formation, which gradually invalidate the original calibration parameters [185,186,187,188,189]. As a result, data semantics across sensing channels are difficult to unify, and signals from different modalities cannot be directly compared or fused. In addition, human–machine co-adaptation is a slow process. The user’s brain must learn how to integrate novel stimulation patterns from artificial devices [190,191], and the sensing–stimulation system must, in turn, adapt its encoding strategies and stimulation thresholds via machine learning, based on physiological feedback [192,193]. Together, these factors make real-time, reliable multimodal fusion a major obstacle to further system optimization.

At a deeper level, current multimodal heterogeneous sensing frameworks lack unified data semantics and interface standards, as well as robust mechanisms for cross-modal fusion and self-calibration. This makes temporal–spatial alignment and uncertainty quantification across channels and devices extremely difficult [187,194]. During long-term operation, the statistical properties of different modalities are nonstationary and exhibit strong inter-individual variability. Static calibration and fusion models cannot cope with these changes. The core bottleneck lies in the absence of a robust cross-modal fusion and automatic calibration pipeline that can operate under multi-rate sampling, time-varying noise, and uncertain delays. Typical limitations include unmodeled delays, covariance mismatch, and domain shifts across users and environments, all of which reduce the potential benefits of multi-source information complementarity. In summary, achieving truly real-time, adaptive, and cross-modal sensing fusion remains a central technical challenge.

#### 4.1.2. Challenge 2: Bandwidth and Latency Limits of In-Body Information Transfer

Current human–machine interfaces are far behind biological neural pathways in terms of information throughput and real-time performance. A key reason is that invasive devices can only provide a limited number of channels and sampling rates. These constraints arise from fabrication limits and strict safety requirements on power and charge injection [8]. As a result, it is difficult to approach the scale of millions of neurons communicating in parallel in the brain. This limitation directly caps the achievable perceptual resolution and control accuracy. For example, the image resolution provided by existing visual prostheses is still extremely low and far from normal vision [9].

Wireless data links between implanted and external units introduce additional bottlenecks. Available bandwidth is limited and link stability is imperfect, with a non-negligible risk of packet loss [195]. For applications that require fast closed-loop feedback, these constraints can become critical [196]. Signal transmission across multiple devices also introduces nontrivial latency, which hinders immediate responses [197]. When several sensing and stimulation modules must operate in coordination, delays at each stage accumulate and may destabilize the control loop. In severe cases, such delay-induced effects can even lead to positive-feedback oscillations.

#### 4.1.3. Challenge 3: System-Level Power Supply and Thermal Management

Fusion of lifeform systems are expected to operate continuously over long periods, yet powering and cooling implanted devices remains a major challenge [12]. Implantable batteries are constrained by limited volume. Their energy capacity and lifetime are therefore restricted, and frequent surgical replacement is clearly undesirable. Wireless power transfer is a promising alternative, but coupling efficiency and energy absorption in biological tissue limit its ability to supply multiple in-body nodes with stable, sufficient power [13].

Many high-performance functions, such as high-speed wireless communication and high-density signal acquisition, are associated with substantial power consumption [198,199]. This power leads to device heating. If the generated heat cannot be dissipated effectively, local tissue temperature may rise to damaging levels. The allowable power density in biological tissue is generally on the order of 80 mW/cm^2^ [200]; so, the temperature rise caused by implanted devices must be strictly controlled. Wireless telemetry is one of the most energy-hungry subsystems [201,202]. Radio-frequency coils used for power and data transfer can cause tissue heating, and their size strongly affects coupling efficiency [203]. When coils are miniaturized to fit into constrained anatomical spaces, energy-transfer efficiency decreases sharply. To meet power demands, the external transmitter must then operate at higher power, which further increases the risk of heating [204].

The internal human environment also imposes severe mechanical and material constraints. Available space is limited, and surrounding tissues are soft and curved. Power-delivery components therefore need to be highly miniaturized, mechanically compliant, and made from biocompatible materials. For devices designed for short- or medium-term use, it is preferable that the power module and other components be fully bioresorbable after completing their function so that no second surgery is needed for removal.

At present, implanted power systems struggle to meet simultaneous requirements on power level, temperature control, and device volume. Balancing these trade-offs is difficult. Enhancing power delivery capability while limiting heat generation and satisfying stringent implantation constraints constitutes a major bottleneck. This challenge calls for new energy-harvesting, storage, and thermal management strategies tailored to long-term in vivo operation.

#### 4.1.4. Challenge 4: Tension Between Biocompatibility and Long-Term Reliability

Long-term implantation of artificial devices inevitably triggers biological reactions that degrade performance over time. The first issue is foreign-body response and tissue encapsulation. Electrodes and sensors in the body are often surrounded within weeks by fibrotic capsules or glial scar tissue [10,11]. This increases impedance and weakens signal transfer. Recorded signals gradually decline, and the current threshold required for stimulation increases [205,206,207]. The central nervous system and peripheral tissues differ in how they respond, but both tend to isolate implants through gliosis or fibrosis, which reduces device effectiveness [208].

Mechanical mismatch and micromotion-induced damage are additional long-term factors. Rigid electrodes have a much higher elastic modulus than surrounding soft tissues. Daily movements then generate small but repeated injuries and chronic inflammation. Micromotion of the implant can also tug on leads, loosen electrode contact, or even cause fractures [209,210]. At the same time, each component of an implanted device has its own failure modes. If the encapsulation layer loses integrity, body fluids can penetrate and cause short circuits and corrosion [211,212]. Electrode materials may dissolve or wear due to long-term electrochemical reactions [213,214]. Leads subjected to tens of thousands of bending cycles can undergo metal fatigue and break [215].

These problems have been repeatedly observed in long-term clinical use and significantly limit the service life and reliability of current implants. Researchers are trying to mitigate these conflicts through advances in materials and fabrication. Examples include ultra-flexible electrodes [216], bioresorbable devices [217,218,219], and improved encapsulation and surface coatings to enhance biological stability [220]. However, building deeply integrated systems capable of stable in vivo operation for decades will require fundamentally new solutions to chronic material–tissue interface compatibility.

#### 4.1.5. Challenge 5: Safety and Reliability in Complex Fusion Systems

When multiple artificial devices operate as a network inside the body, system complexity increases dramatically [221,222], and ensuring reliability becomes extremely difficult. Devices may interfere with each other electromagnetically, especially when several wireless modules are active at the same time. Sensing loops and stimulation loops must also be designed to avoid mutual interference [223]. In complex architectures, cascading failures are more likely. A malfunction in any single subsystem can degrade overall performance or, in extreme cases, endanger the user’s life [224].

At present, there is no unified set of interface standards or communication protocols for such human–machine fusion systems. Devices developed by different groups are hard to interconnect or interoperate. This lack of standardization limits large-scale deployment and long-term upgradability. Evaluation benchmarks for multimodal fusion systems are also missing. Traditional performance metrics, such as improvement in a single-organ function, cannot fully capture the overall effect of multimodal coordination. Cross-domain behavioral assessment methods are still not standardized. As a result, outcomes from different experimental platforms are difficult to compare or reproduce, which slows technical iteration and clinical translation.

Potential safety risks add further complexity [225,226]. Runaway behavior in closed-loop systems is a major concern. If a sensor reports incorrect values or a control algorithm makes an erroneous decision, continuous automatic stimulation may overcompensate a physiological parameter and cause new instabilities. For example, failure in closed-loop control of an insulin pump can lead to dangerous hypoglycemia [227]. Fusion systems therefore need built-in redundancy checks and safety thresholds. When abnormal states are detected, the system should enter a safe mode, shut down stimulation, or issue an alarm.

As implants become networked and more interconnected, cybersecurity also becomes critical. Devices may be vulnerable to hacking or unauthorized modification, with potentially severe consequences. Ensuring the safety and reliability of such complex hybrid bio–machine systems will require comprehensive governance mechanisms. This is one of the key challenges that will determine whether fusion of lifeform technologies can ultimately earn trust and achieve large-scale deployment.

### 4.2. Future Directions

#### 4.2.1. Building Layered, Heterogeneous In-Body Intelligent Communication Networks

To address the information-transfer bottlenecks discussed in Section 4.1, future interface systems are likely to develop along two parallel communication paths: high-speed short-range optical links, and low-loss ultrasound links for deep or distant tissues. These two modalities are complementary and can jointly support an in-body high-speed network.

For optical communication, studies have shown that near-infrared links inside the body can markedly increase data rates. Light experiences relatively low absorption and electromagnetic interference in biological tissues, which allows for more efficient transmission [228]. Recent experiments in tissue-mimicking media have demonstrated data rates above 100 Mbps over sub-centimeter distances [123,229]. Data rates on the order of 100 kbps have also been achieved over sub-decimeter links [230]. In the future, miniaturized semiconductor laser or LED arrays, combined with modulation schemes such as wavelength-division multiplexing, may further expand bandwidth [229]. This would enable high-speed, short-range optical links between dense clusters of implants and allow for “optical routing” nodes inside the body that aggregate data from multiple sensors and forward these to a subcutaneous relay. To mitigate scattering and improve channel stability, transparent cranial windows [231,232,233] or refractive-index-matched implant materials [234,235] could provide stable optical paths.

For longer distances across organs or to deeper regions, ultrasound is more advantageous. Ultrasound attenuates much less than RF waves in tissue and can also be used for power transfer. This makes it a strong candidate to replace RF for in-body backbone communication [236,237]. Proof-of-concept studies have already achieved data rates above 30 Mbps through about 5 cm of tissue and have shown that the same ultrasonic link can deliver both data and power [238]. Future work may employ array-based ultrasonic transducers with spatial multiplexing to support parallel communication with multiple nodes [239,240]. New waveguide structures and epidermal acoustic channels based on flexible materials could act as in-body or on-skin conduits for ultrasound, enabling low-loss communication over tens of centimeters without increasing tissue damage [239,241].

In such architectures, high-speed optical links would handle real-time data exchange among local high-density nodes, while ultrasound would form a whole-body backbone network. Joint design of modulation and coding schemes, beamforming control, and routing protocols would allow for seamless switching and fusion between the two media. The resulting infrastructure could provide the “high bandwidth + low latency + wide coverage + low power” profile required by fusion of lifeforms. It would support coordinated operation of multimodal devices, help solve cross-modal data-synchronization issues highlighted in Challenge 1, and improve overall system reliability relevant to Challenge 5.

#### 4.2.2. Sustainable and Adaptive Power and Thermal-Management Strategies

The power and thermal challenges outlined in Challenge 3 ultimately arise from physical limits on energy density and heat dissipation. A promising direction is to develop sustainable and adaptive in vivo power and thermal management strategies. In such systems, multiple energy sources and cooling techniques are integrated, and power delivery is dynamically adjusted according to physiological state, forming an energy framework that “coexists” with the host.

On the supply side, one goal is to improve the efficiency and safety of implanted wireless power transfer [242]. Magnetic resonant coupling and ultrasound-based power delivery are two representative approaches. They can provide continuous energy to multiple implanted nodes without interrupting surrounding tissues. In parallel, in vivo energy harvesting can partially support self-powered operation of implants. Glucose biofuel cells are a particularly attractive option. Studies have shown that a single implanted enzymatic fuel cell can continuously harvest tens of microwatts from glucose in body fluids. This is sufficient to light an LED or drive small sensors and can operate in rats for several months without obvious immune rejection [243]. Other techniques, such as motion-energy harvesting [244,245,246] and thermoelectric generation [247], can tap into endogenous sources like heartbeat, respiration, or muscle contraction, thereby reducing dependence on external power.

Thermal management must be advanced in parallel to control temperature rise during device operation. One approach is to integrate phase-change materials into the implant as thermal buffers. When the device heats up, these materials absorb latent heat and smooth temperature peaks [248,249]. Another option is to design microfluidic cooling paths that conduct heat away from hot spots toward larger surfaces where it can dissipate more safely [250,251]. Experimental data suggest that as long as power density is kept below about 80 mW/cm^2^ [200], irreversible tissue damage can be avoided, making precise thermal control essential.

Finally, the power-supply and heat-management components of the implant must still meet strict requirements for miniaturization, mechanical flexibility, and biocompatibility. This includes encapsulating power components in soft, biocompatible materials so that the system can bend and deform with body movements without injuring tissue. With these advances, future fusion of lifeform systems may employ intelligent power modules that adjust output dynamically to the internal environment, provide sufficient energy, and actively suppress overheating, thereby greatly improving both endurance and safety.

#### 4.2.3. Innovative Biointerfaces and Long-Lived Encapsulation Materials

To address the fundamental tension between biocompatibility and long-term reliability (Challenge 4), future work must advance both biointerface materials and encapsulation technologies. From a material science and bioengineering perspective, new materials and structural designs can improve compatibility between artificial devices and living tissue at the source. This, in turn, can significantly extend the safe service life of implants.

One key direction is the development of flexible electronic materials with mechanical properties that match those of human tissues. Soft substrates and stretchable conductors allow implanted devices to deform together with surrounding tissue [252,253,254,255,256]. In particular, advanced fabrication techniques such as electrospinning enable the scalable production of highly conformable, breathable nanofibrous membranes that further enhance mechanical compliance and long-term biocompatibility in both wearable and implantable systems [256]. This reduces cutting and friction damage caused by rigid materials. Mesh-like flexible electrodes have already shown great potential in brain implants by markedly attenuating immune responses and maintaining stable signals over long periods [216,257]. By lowering the effective Young’s modulus and optimizing the geometric design, flexible electronics can minimize chronic inflammation and scar formation induced by mechanical mismatch.

A second direction is the use of bioresorbable materials, which offers a fundamentally different design philosophy. Transient electronic devices are built from polymers such as polyanhydrides, polylactic acid (PLA), poly(lactic-co-glycolic acid) (PLGA), and silk fibroin, together with dissolvable metals and semiconductors [219,258,259,260]. After a predefined service period, the device gradually dissolves in body fluids and is cleared by normal metabolic pathways, eliminating the need for surgical removal. At the device level, resorbable silicon electronics have been used in animal brains for multimodal physiological monitoring and then disappeared after the task [261,262]. Fully bioresorbable wireless temporary cardiac pacemakers have demonstrated a truly leadless, battery-free, and fully cleared implant pathway. These examples provide strong in vivo evidence for “use-and-disappear” clinical strategies [263]. Overall, this approach combines chemical composition, geometry, and encapsulation design to program device lifetime. The system retires itself before performance degrades severely and is safely processed by the body. This avoids risks associated with long-term implantation, such as material aging, barrier failure, and chronic inflammation, and supports regulatory evaluation through well-defined lifetime–degradation curves and traceable safety profiles of degradation products.

Drug-eluting surface coatings offer another effective means to improve interface compatibility. Anti-inflammatory agents or neurotrophic factors can be loaded onto electrode surfaces and released gradually during the early post-implantation period. This helps suppress acute inflammation and glial scarring, protects neurons near the electrode, and reduces signal loss and tissue damage [218]. In parallel, new encapsulation technologies are being developed, including multilayer flexible barriers and superhydrophobic or anti-biofouling coatings [220]. These designs aim to increase resistance to body-fluid ingress and mechanical fatigue. By lowering the risk of cracking, delamination, and leakage, such encapsulation strategies help ensure that internal electronic components continue to operate reliably in complex in vivo environments.

Through a combined strategy that couples advances in materials, structural design, and pharmacology, future implantable electronics may achieve both high performance and markedly improved biocompatibility and durability. Flexible electronics can reduce tissue stress; drug coatings can mitigate immune responses; degradable materials can eliminate long-term foreign-body residues and revision surgery; and robust encapsulation can protect the remaining electronics over extended periods. Together, these innovations will extend the lifetime of fusion systems, reduce complications and maintenance needs, and make long-term human–machine integration a realistic goal.

#### 4.2.4. Frameworks for System-Level Safety and Reliability

Beyond improving the reliability of individual devices, the safety of the entire fusion system is even more critical. This calls for a system-level safety and reliability framework that provides dual protection analogous to an “immune system” and a “nervous system” for fusion of lifeforms. The “immune” layer would detect and contain local faults through self-diagnosis and fault-tolerant mechanisms, preventing cascading failures. The “neural” layer would consist of secure communication protocols and global control strategies that coordinate all modules and ensure that information exchange remains reliable, efficient, and controlled.

In practice, robust fail–safe mechanisms are needed. Critical sensors and actuators should be deployed with redundancy so that backup units can take over seamlessly if one fails. Real-time anomaly detection algorithms should monitor deviations from normal operating ranges and trigger early warnings. Layered safety shutdown strategies are also required. When risk indicators are detected, the system should automatically downgrade functionality or shut down specific modules to prevent harm to the user. In parallel, cybersecurity defenses must be significantly strengthened. Encrypted communication, authentication, and intrusion detection techniques are essential to prevent implanted devices from being controlled by unauthorized external commands. Even in open, networked environments, such measures help maintain the confidentiality and integrity of internal signals and protect patients against malicious attacks.

Digital twin technology can serve as a powerful tool to enhance reliability and safety [264,265]. By creating a virtual replica of the in-body fusion system and its interaction with the physiological environment, engineers can simulate potential failure modes, interface issues, and extreme conditions before real-world deployment. Digital twins can also be used to repeatedly test control algorithms and fault-handling strategies under a wide range of abnormal scenarios, thereby optimizing fault tolerance and emergency responses and reducing real-world risk. During actual operation, the digital twin can function as a real-time monitoring and decision support layer that flags emerging anomalies and suggests corrective actions, improving autonomous safety management. At the ecosystem level, standardized interfaces and evaluation frameworks are urgently needed.

Common hardware interface specifications and communication protocols should be established to address cross-modal semantic and interface incompatibilities highlighted in Challenge 1, and to ensure that modules from different sources are plug-and-play and interoperable. A comprehensive metric set is also required. It should cover multimodal coordination performance, biocompatibility, fault recovery capacity, and other system-level properties so that solutions from different groups can be compared on a common basis. Regulatory bodies and the academic community will need to co-develop certification standards and ethical guidelines tailored to fusion of lifeform systems, providing clear safety boundaries and regulatory frameworks for clinical translation. Only when architecture design, fault protection, simulation-based validation, and industry-wide standards advance together can the safety and reliability of fusion of lifeforms be robustly guaranteed, enabling complex human–machine integration to evolve into a trustworthy, mature technology.

While overcoming the engineering barriers outlined in Section 4 is essential for the physical realization of fusion of lifeforms, the very act of solving these challenges—such as enabling high-bandwidth neural links and increasingly autonomous closed-loop systems—also amplifies their ethical and societal ramifications. System-level complexities in interoperability, security, and safety do not exist in a vacuum; they directly translate into risks of neural privacy breaches, erosion of human agency, and ambiguity in legal responsibility. Consequently, efforts to address technological bottlenecks in integration and control must be accompanied by a proactive and rigorous framework for ethical oversight and governance, as explored in the following section.

## 5. Safety and Ethical Considerations

Fusion-life technologies give rise to a series of cutting-edge ethical challenges. The first concerns blurred self-identity and shifting boundaries of personhood. Brain–computer interfaces and related technologies extend the scope of human identity, making cyborg-like human–machine hybrids possible; yet, while they may benefit humanity, they inevitably introduce high levels of risk and thus draw increasing ethical scrutiny. When thoughts and perceptions become deeply integrated with machine systems, individuals may experience philosophical uncertainty about “who I am”: if artificial components reshape cognition and personality, is the original self still intact? Case reports suggest that long-term implanted devices can indeed affect self-perception; some patients, for example, have wept when forced to have their implanted BCI removed, stating that “I lost myself” [266]. Some scholars argue that the development of enhanced BCIs (eBCIs) has moved beyond a simple cost–benefit calculus and now compels us to reconsider the nature of conscious selfhood and to ask who we are—and who we ought to become—in a fused human–machine condition [267]. At the same time, autonomous decision-making and free will face potential erosion. If neural devices can independently modulate emotion or steer decisions, to what extent does the user remain fully autonomous? Ethicists warn that “If you have a device that constantly steps up in your thinking or decision-making,” as Gilbert notes, “it might compromise you as an agent” [266]. In practice, people tend to defer to technologically superior recommendations: “You have the ultimate decision,” Gilbert observes, “but as soon as you realize the device is more effective in the specific context, you won’t even listen to your own judgement. You’ll rely on the device.” This phenomenon suggests that, within a cyborg mind co-governed by human and machine, the boundary between personal autonomy and system intelligence is becoming increasingly blurred.

Second, neuroprivacy and mental security present major ethical concerns. BCIs can directly read and influence brain signals, exposing an individual’s inner mental world to unprecedented exposure risk. The information within the brain is arguably the most intimate and personal form of data. If neural signals are improperly accessed, personal thoughts could be “read” or even manipulated. These risks of data misuse and “mind-reading” have spurred calls for neuroprivacy to be legally protected, akin to bodily privacy, with theoretical proposals advocating for “neurorights”—including freedom of thought, privacy, mental integrity, and personal identity—to safeguard mental sovereignty [268]. Meanwhile, issues of fairness and ethical inequality also arise. The high cost of cognitive enhancement devices may restrict access to a privileged few, potentially worsening social inequity. Concerns exist that if only the wealthy can obtain enhanced BCIs, the conferred cognitive advantages could widen pre-existing socioeconomic disparities. This may lead to a societal split between “enhanced” and “natural” individuals, creating new unfair competition in areas like education and employment. Such a cognitive divide challenges social equity and risks provoking new forms of discrimination and social tension.

Finally, fusion-life systems pose unprecedented challenges for legal responsibility and ethical governance. When human–machine hybrid systems participate in behavioral decisions or even act autonomously, assigning responsibility becomes complex and ambiguous. Consider an accident involving an exoskeleton with AI-based co-decision capabilities: should liability fall on the human operator, on the machine intelligence, or be shared between them? The existence of such hybrid agents can create a “responsibility vacuum,” as part of the decision-making originates in the human user and part in machine algorithms. Current legal frameworks typically treat machines as tools and humans as the sole bearers of responsibility, and have not yet formally recognized human–machine hybrids as a distinct, intermediate category. Ethical oversight and legal structures therefore need urgent updating. Regulators and researchers must develop clear guidelines and responsibility allocation mechanisms tailored to this new composite form of life, ensuring that society is adequately prepared to address the ethical and legal challenges before these technologies are deployed at scale.

## 6. Conclusions

This review synthesizes multi-level perception systems within the concept of fusion of lifeforms, defined as long-term symbiotic integration of biological and artificial components across structural, energetic, informational, and cognitive axes. We propose a four-class functional taxonomy—sensory restoration, beyond-natural sensing, endogenous state monitoring and regulation, and cognitive enhancement—and survey-representative technologies spanning neuroprostheses, implantable sensors, and brain–computer interfaces, highlighting a shift from external aids toward embedded, co-adaptive hybrid systems. Key bottlenecks include multimodal fusion, constrained in-body bandwidth and power, long-term biocompatibility, and the absence of unified interface standards and outcome metrics. Progress will require advances in materials, energy harvesting, in-body communication, and AI-driven decoding and closed-loop control, alongside fail–safe design principles. Finally, the convergence of human and machine intelligence demands parallel attention to identity, neural privacy, equity, and accountability.

## Figures and Tables

**Table 1:** Key studies systematic comparison across Class I–II perception systems.

Ref (Year)	Class/Function	Interface and Site	Key Outputs/Outcomes	Main Limitation/Bottleneck	Key Sensor Metrics
[20] (2024)	I/Auditory restoration	Fully Implantable CI	In vivo guinea pig validation: frequency-selective stimulation; eABR evoked for ∼45–100 dB SPL	Weak off-resonance/band-edge sensitivity; packaging needed; power–range trade-off	8-ch MEMS; ∼300 mVpp@100 dB; <600 μW
[21] (2024)	I/Auditory restoration	Noise reduction technology of CI	Significant improvement in multi-talker speech-in-noise perception	High computational cost; not yet fully implantable in real time	20% intelligibility (+5 dB SNR); 22 channels; high stability; low-latency RNN
[22] (2016)	I/Visual restoration	Epiretinal electrode array (retina)	Stable perception of light and spatial location over 5 years	Extremely low spatial resolution; reliance on external camera	60 electrodes ( 20/1260 acuity); chronic 5+ years; wireless inductive low mW.
[23] (2015)	I/Visual restoration	Subretinal photodiode array (retina)	Higher visual acuity than epiretinal systems in preclinical studies	Limited field of view; requires external IR projector	70 μm pixels; preclinical acute; photovoltaic IR low power.
[24] (2023)	I/Speech restoration	Intracortical microelectrodes (motor cortex)	Real-time speech synthesis up to ∼62 words/min in paralyzed patients	Invasive interface; limited vocabulary size and long-term stability	62 wpm speech; 23.8% WER; 80 ms latency; 128 electrodes
[25] (2018)	I/Tactile restoration	E-skin sensors with peripheral nerve interface	Restored pain sensations and enabled discrimination of object curvature and sharpness via the prosthesis	Single-subject demonstration; coarse and non-natural sensations	0–300 kPa range; graded touch-pain; 3 taxels/fingertip; multilayer higher epidermal sensitivity
[26] (2019)	I/Tactile restoration	Peripheral nerve electrodes (upper limb)	Biomimetic sensory feedback via nerve stimulation improved dexterous bionic hand control and embodiment	Study limited to a single participant	Contact force/torque sensors: 0–25.6 N range, 0.1 N/bit, 30 Hz.
[27] (2025)	I/Olfactory restoration	Olfactory bulb interface	Induced smell perception via olfactory bulb electrical stimulation	Small sample (*n* = 5); subjective reports without objective confirmation	Induced smell (3/5 subjects); 1–20 mA, 3.17 Hz; subjective perception; small-sample.
[28] (2019)	II/Cardiac sensing	Implantable TENG pressure sensor (ventricle)	Self-powered ultrasensitive sensor enables real-time endocardial pressure monitoring	Limited to animal testing; durability and chronic stability unclear	Self-powered; 1.195 mV/mmHg sensitivity; R^2^ = 0.997; 0–350 mmHg; 10^8^ cycles
[29] (2021)	II/Cardiac sensing	Gapless TENG sensor (myocardium)	No-spacer TENG enables precise cardiac monitoring	Preclinical testing in animal model	Self-powered; 3.67 V Voc, 51.7 nA Isc, 99.7% HR, $10^{6}$-cycle stable
[30] (2022)	II/Cardiac regulation	TENG-based stimulation interface (myocardium)	Self-triggered pacing improves cardiac function in animal models	Insufficient output energy for large-scale or human application	Self-powered TENG; 0.4–20 V, 20–80 V/cm EF, 100–400 µm depth
[31] (2018)	II/Metabolic regulation	TENG sensor with vagus nerve interface	Closed-loop appetite suppression and weight reduction in rats	Unknown long-term biocompatibility; invasive implantation	Battery-free; 0.05–0.12 V pulses, 12-week stable, 40 µW
[32] (2018)	II/Urinary control	TENG sensor with SMA actuator (bladder wall)	Autonomous on-demand bladder voiding in underactive models	Early feasibility stage; limited lifespan of actuatorsq	Output 35.6–114 mV for 0–6.86 N; saturates 0.67 mL
[33] (2019)	II/Orthopedic therapy	TENG electrodes at fracture site	Enhanced osteogenesis and bone healing in osteoporotic rats	Low stimulation power; preclinical validation only	TENG 100 V, 1.6 μA; EF 150 V/cm, 250 μm

**Table 2:** Key studies’ systematic comparison across Class III–IV perception systems.

**Ref (Year)**	**Class/Function**	**Interface and Site**	**Key Outputs/Outcomes**	**Main Limitation/Bottleneck**	**Key Sensor Metrics**
[156] (2012)	III/Geomagnetic sense	Vibrotactile belt (waist skin)	Users developed a “sense of north”; improved navigation/orientation tasks	Requires long training; limited information bandwidth (direction only)	Pointing error 41 → 23°, 163 → 84°; walk deviation 10°
[157] (2013)	III/Infrared sense	Intracortical microstimulation (S1 cortex, rat)	Rats learned to detect IR signals; new IR perception coexisted with normal touch	Invasive animal implant; simple stimulus representation (single-pixel IR)	IR prosthesis: ICMS 0–400 Hz, 93% correct, 1.3 s
[158] (2021)	III/Infrared sense	Retinal prosthesis input fusion (Argus II)	Improved night navigation and human detection for prosthetic vision users	Additional external hardware; low-resolution thermal overlay	Thermal camera: 60 electrodes; $11^{\circ }\times 19^{\circ }$ FOV ( 22° diagonal); 200 μm diameter
[159] (2015)	III/Echolocation	Head-mounted ultrasonic sensor + stereo audio	Users learned to judge object distance/direction via sound after training	Training-dependent; limited spatial resolution and throughput vs. natural vision	25–50 kHz bandwidth; 160° microphone field of view; echoes to 5 m; 75–86% correct
[160] (2025)	IV/BCI skill learning	EEG headset (scalp)	Improved motor-imagery BCI accuracy via co-adaptive neurofeedback training	non-invasive signals limit resolution; gains can be task-specific	62 electrodes; 512–1000 Hz sampling; 0.5–40 Hz bandwidth.
[161] (2025)	IV/Cognitive therapy	Transcranial ultrasound (head)	Reported cognitive-score improvements and increased brain network activity vs. sham	Mechanism unclear; small cohort and transient effects	$0.20\;{\mathrm{mJ}/\mathrm{mm}}^{2}$ flux; 5 Hz frequency; 3 μs duration1.
[162] (2018)	IV/Memory enhancement	Cortical electrodes (temporal lobe)	Improved word recall with adaptive, timed stimulation in epilepsy patients	Invasive; variable benefit across individuals and tasks	3–180 Hz bandwidth; 0.61 AUC; OR 1.18 (recall)
[163] (2017)	IV/Memory enhancement	Depth electrodes (hippocampus, primate)	Improved memory-task performance using closed-loop hippocampal pattern stimulation	Highly invasive; demonstrated only in animal models with external computing	10–50 μA current; 1.0 ms pulses; ≤ 20 Hz frequency; 70–75% accuracy.
[164] (2025)	IV/Memory enhancement	Depth electrodes (hippocampus, human)	Enhanced hippocampal network connectivity associated with memory-related function	Invasive; cognitive benefits not yet fully quantified	30 kHz sampling; 5–10 mm spacing; 0.1–1 kHz bandwidth; 500 Hz rate

## Data Availability

No new data were created or analyzed in this study. Data sharing is not applicable to this article.

## Abbreviations

The following abbreviations are used in this manuscript:

AGI	Artificial General Intelligence
ALS	Amyotrophic Lateral Sclerosis
B2BI	Brain-to-Brain Interfaces
BBIs	Brain-to-Brain Interfaces
BCI	Brain–Computer Interfaces
CBIs	Computer–Brain Interfaces
CGM	Continuous Glucose Monitoring
DBS	Deep Brain Stimulation
ECG	Electrocardiogram
EEG	Electroencephalography
eBCIs	Enhanced Brain–Computer Interfaces
EMG	Electromyography
e-nose	Electronic Nose
FNIRs	Functional Near-Infrared Spectroscopy
FUS	Focused Ultrasound
ICMS	Intracortical Microstimulation
iBCIs	Implantable Brain–Computer Interfaces
iEEG	Intracranial Electroencephalography
IMU	Inertial Measurement Units
IoB	Internet of Bodies
MIMO	Multi-Input–Multi-Output
NSTENG	Nano-Structured Triboelectric Nanogenerator
PLA	Polylactic Acid
PLGA	Poly(lactic-co-glycolic acid)
SEPS	Subendocardial Pressure Sensors
SSVEP	Steady-State Visual Evoked Potentials
TENGs	Triboelectric Nanogenerators
TENS	Transcutaneous Electrical Nerve Stimulation
TMS	Transcranial Magnetic Stimulation
WIBSNs	Wearable–Implantable Body Sensor Networks
